# Liver macrophage-associated inflammation correlates with SIV burden and is substantially reduced following cART

**DOI:** 10.1371/journal.ppat.1006871

**Published:** 2018-02-21

**Authors:** Bridget S. Fisher, Richard R. Green, Rachel R. Brown, Matthew P. Wood, Tiffany Hensley-McBain, Cole Fisher, Jean Chang, Andrew D. Miller, William J. Bosche, Jeffrey D. Lifson, Maud Mavigner, Charlene J. Miller, Michael Gale, Guido Silvestri, Ann Chahroudi, Nichole R. Klatt, Donald L. Sodora

**Affiliations:** 1 Center for Infectious Disease Research, formally Seattle Biomedical Research Institute, Seattle, Washington, United States of America; 2 Center for Innate Immunity and Immune Disease, Department of Immunology, University of Washington, Seattle, Washington, United States of America; 3 Department of Pharmaceutics, Washington National Primate Research Center, University of Washington, Seattle, Washington, United States of America; 4 Cornell University College of Veterinary Medicine, Department of Biomedical Sciences, Section of Anatomic Pathology, Ithaca, New York, United States of America; 5 AIDS and Cancer Virus Program, Leidos Biomedical Research, Inc., Frederick National Laboratory for Cancer Research, Frederick, Maryland, United States of America; 6 Department of Pathology and Laboratory Medicine, Emory University School of Medicine, Atlanta, Georgia, United States of America; 7 Emory Vaccine Research Center and, Yerkes National Primate Research Center, Atlanta, Georgia, United States of America; 8 Emory University School of Medicine, Department of Pediatrics, Atlanta, Georgia, United States of America; Miller School of Medicine, UNITED STATES

## Abstract

Liver disease is a leading contributor to morbidity and mortality during HIV infection, despite the use of combination antiretroviral therapy (cART). The precise mechanisms of liver disease during HIV infection are poorly understood partially due to the difficulty in obtaining human liver samples as well as the presence of confounding factors (e.g. hepatitis co-infection, alcohol use). Utilizing the simian immunodeficiency virus (SIV) macaque model, a controlled study was conducted to evaluate the factors associated with liver inflammation and the impact of cART. We observed an increase in hepatic macrophages during untreated SIV infection that was associated with a number of inflammatory and fibrosis mediators (TNFα, CCL3, TGFβ). Moreover, an upregulation in the macrophage chemoattractant factor CCL2 was detected in the livers of SIV-infected macaques that coincided with an increase in the number of activated CD16+ monocyte/macrophages and T cells expressing the cognate receptor CCR2. Expression of Mac387 on monocyte/macrophages further indicated that these cells recently migrated to the liver. The hepatic macrophage and T cell levels strongly correlated with liver SIV DNA levels, and were not associated with the levels of 16S bacterial DNA. Utilizing *in situ* hybridization, SIV-infected cells were found primarily within portal triads, and were identified as T cells. Microarray analysis identified a strong antiviral transcriptomic signature in the liver during SIV infection. In contrast, macaques treated with cART exhibited lower levels of liver macrophages and had a substantial, but not complete, reduction in their inflammatory profile. In addition, residual SIV DNA and bacteria 16S DNA were detected in the livers during cART, implicating the liver as a site on-going immune activation during antiretroviral therapy. These findings provide mechanistic insights regarding how SIV infection promotes liver inflammation through macrophage recruitment, with implications for in HIV-infected individuals.

## Author summary

Liver disease is common in HIV-infected individuals and is one of the leading causes of death in this population. Currently, the factors that contribute to liver disease during HIV infection are not known, as human studies are difficult to conduct. Therefore, pathogenic SIV infection of macaques is a valuable model system for understanding immune changes that occur in tissues during infection, including the liver. Using liver samples from uninfected and SIV-infected macaques, we observed an increase in liver macrophage infiltration that was likely mediated by the macrophage-attracting chemokine, CCL2. Importantly, liver macrophage number strongly correlated with liver SIV levels and the expression of liver inflammatory and fibrosis mediators during SIV infection. Further, we identified an upregulation of immune and inflammatory pathways in the liver of SIV-infected macaques, including a strong antiviral response. Treatment with antiretroviral drugs decreased macrophage infiltration and reduced liver inflammation, consistent with reduced SIV levels. In summary, these findings provide mechanistic insights into how SIV infection promotes liver inflammation through macrophage infiltration, which can predispose this population to liver complications during infection.

## Introduction

Liver disease has become a leading contributor to morbidity and mortality in HIV-infected people with the occurrence of nonalcoholic fatty liver disease (NAFLD) being one of the most predominant complications [1–5]. Within the setting of HIV infection, not only is NAFLD more common than in the general population, but also is more severe with higher incidence of steatohepatitis (NASH), liver injury, and lobular inflammation when compared to HIV-negative people [4, 6]. Liver inflammation can directly impact systemic circulation through the production of acute-phase proteins and failure to detoxify gut-derived blood to potentially contribute to the chronic inflammatory conditions commonly observed in HIV-infected people [7–9]. Further, the liver plays a role in the metabolism of antiretroviral drugs, and in some studies NAFLD and fibrosis are exacerbated during combination antiretroviral treatment (cART), and is most often associated with low CD4 T cell counts, alcohol abuse, high immune activation, and exposure to certain ART drugs, such as didanosine [4, 10, 11]. Other studies have identified improvement of liver fibrosis during drug treatment, associated with better recovery of CD4 T cells during therapy and younger age [12]. Liver biopsies are currently considered to be the most precise method for diagnosing hepatic disease, however, this invasive procedure can have complications, such as pain, hemorrhage and sepsis, which make human studies difficult to conduct [13, 14].

Experimental simian immunodeficiency virus (SIV) infection of macaques has provided useful models for studying viral pathogenesis and the host immune response, particularly in tissue compartments. Pathogenic SIV infection of macaques has provided key insights in the pathogenesis of HIV/SIV infection, including identifying the gut as a major site of viral replication and CD4 T cell depletion [15, 16], elucidating the mechanisms of mucosal dysfunction [17–20], and defining viral reservoirs [21–25]. In addition, SIV-macaque models have been utilized to establish the liver as a primary site of SIV clearance *in vivo* [26] and to evaluate liver immune cells during SIV infection, including macrophages, T cells, and NK cells [27–29]. Indeed, one study found that some of the T cells that infiltrate the liver during infection are SIV-specific CD8 cells that localize to the portal triad regions of the liver [30], the area where the portal vein, hepatic artery and bile duct converge. The portal vein, which drains the gastrointestinal tract, gall bladder, pancreas and spleen, is of particular interest as in contrast to other veins, it does not conduct blood back to the heart, but rather supplies the hepatic capillary beds, bringing nutrients and ingested toxins to the liver for processing. This unusual circulatory pattern also provides an opportunity for microbial products from the gastrointestinal tract to enter the liver, especially when intestinal epithelial integrity is compromised. Liver dysfunction leads to incomplete clearance of bacterial products from the blood, and increased presence of translocated microbial products in systemic circulation, which correlates with immune activation/inflammation in SIV-infected macaques [31, 32].

The exact mechanisms of liver disease pathogenesis during HIV/SIV infection are yet to be fully defined. It was previously discovered that CXCL16 production induces NK cell infiltration into the liver during SIV infection [29] supporting a role for chemokines in promoting liver disease during infection. Indeed, chemokine-associated immune cell infiltration has been implicated in a spectrum of liver diseases, including viral hepatitis, fibrosis, and alcoholic liver disease [33]. In particular, the CCL2-CCR2 axis is critical for the progression of acetaminophen-induced hepatotoxicity [34], fibrosis [35], and steatohepatitis [36, 37] through the recruitment of hepatic macrophages. Disruption of this chemokine network reduces both hepatic macrophage number and associated liver pathologies [36, 38, 39] highlighting the central role that infiltrating hepatic macrophages can play in liver disease.

The goal of this study was to delineate the immunologic and inflammatory factors that contribute to liver disease progression during retroviral infection. As liver disease develops over many decades, this cross-sectional study focused on the early mediators that trigger subsequent hepatic dysfunction, and included untreated adult and infant macaques that were infected with SIV, as well as macaques that were SIV-infected and receiving cART. We report that SIV levels in the liver are associated with macrophage infiltration and hepatic T cell numbers. Transcriptomic analysis revealed an inflammatory signature in the livers of SIV-infected macaques that was dominated by a strong antiviral response that was diminished in the livers from the cART treated macaques. These findings provide critical mechanistic insights regarding how SIV infection impacts liver inflammation and viral replication, which will be important for designing therapies to ameliorate liver complications during HIV infection.

## Results

### Quantification of liver T cells and macrophages

**Fig 1:**
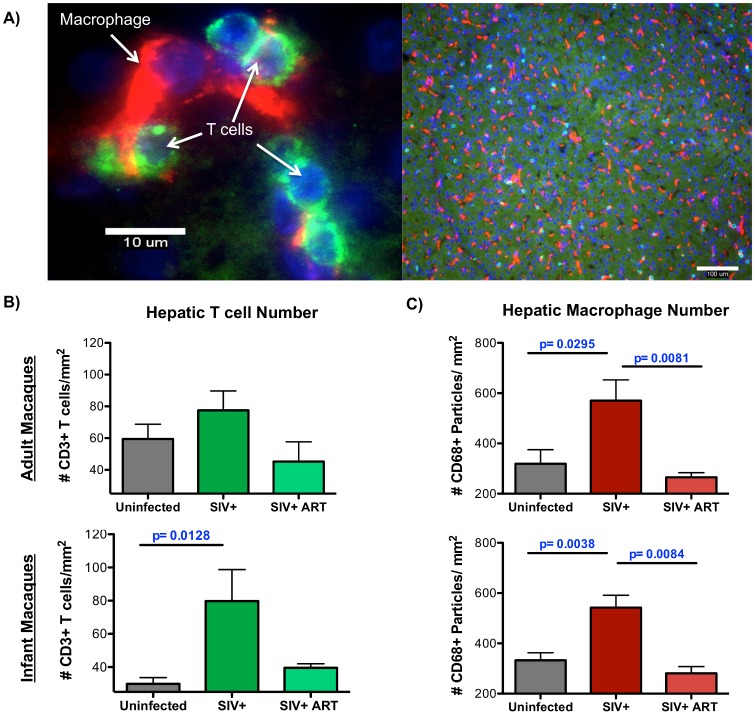
Increased number of macrophages and T cells observed in the liver during SIV infection. CD68 macrophages and CD3 T cells were enumerated in the liver of uninfected, chronically SIV-infected, and chronically SIV-infected, cART infant or adult rhesus macaques by immunofluorescence microscopy. **A)** Fluorescent images at 600x (left image, scale bar = 10 um) and 100x (right image, scale bar = 100 um) depicting specific staining for macrophages (red) and T cells (green) in the liver (blue indicates nuclei). For quantification, eight random fields in the liver were imaged under 100x magnification and then analyzed by ImageJ software. **B)** Quantification of eight random fields of view for each animal to enumerate CD3 T cells by ImageJ Cell Counter analysis with adult and infant macaques graphed separately (top and bottom panels, respectively). **C)** Quantification CD68 macrophages in eight random fields of view for each animal using ImageJ Particle analysis. Data are graphed as the mean ± SEM. Statistical significance between groups was determined Mann Whitney T tests.

During the development of liver disease, the presence of different immune cell subsets provides information regarding the immune cell populations that drive liver inflammation while the intrahepatic localization of these immune cells determines the nature of disease [27, 28]. Utilizing immunofluorescence microscopy, macrophages and T cells were quantified in the liver using cell-specific CD68 and CD3 staining, respectively (Fig 1A). Although elevated numbers of macrophages and T cells, both CD4 and CD8 T cells, were observed in the portal regions during SIV infection (S1 Fig), there was noticeable variation in the levels of immune cells observed within each liver section of infected macaques. Therefore, these quantitative analyses focused on lobular regions of the liver without any portal triads. In SIV-infected infant macaques, a significant increase in T cells was observed compared to uninfected infants. A trend for increased T cells in SIV-infected adult macaques was also observed, although this difference did not reach statistical significance (Fig 1B). Likewise, the number of liver macrophages was also significantly increased in both infants and adults during SIV-infection (Fig 1C). In macaques that were receiving cART, the levels of both liver T cells and macrophages were reduced to levels comparable to uninfected macaques suggesting that this immune cell infiltration was reversible when SIV replication is suppressed.

### Assessment of liver CCL2 and CCR2 levels

**Fig 2:**
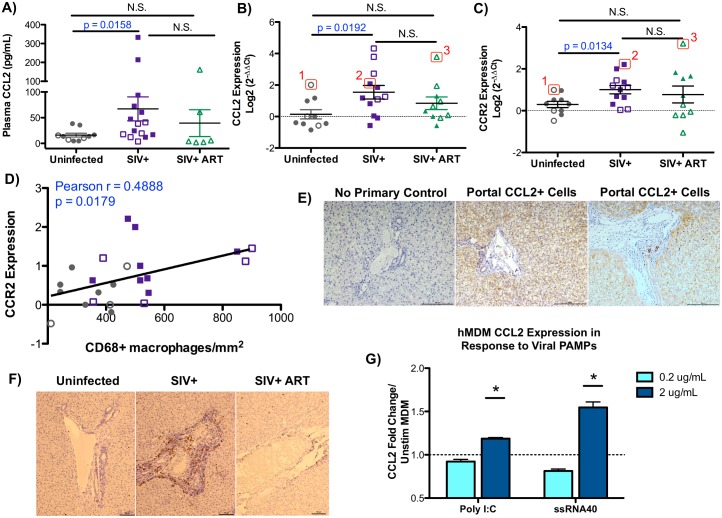
The CCL2-CCR2 pathway is associated with liver monocyte/macrophage infiltration. **A)** Levels of CCL2 (MCP-1) in the blood were determined by Luminex analysis. Data are graphed as the mean ± SEM. Adult (open symbols) and infant (closed symbols) macaques are denoted in each graph. **B-C)** Hepatic expression of CCL2 (B) and its receptor, CCR2, (C) were evaluated by quantitative PCR using cDNA prepared from RNA isolated from the liver of each animal. Data are graphed as log2 transformed relative expression levels determined by the comparative threshold method. Numbered macaques (1, 2, and 3) denote CCL2 and CCR2 expression levels for these three individual macaques between panels 2B and 2C. **D)** Correlation between the expression of CCR2 in the liver (y-axis) and the number of liver macrophages (x-axis) in uninfected (gray points) and SIV-infected (purple points) macaques was evaluated by Pearson Correlation analysis. **E)** Immunostaining for CCL2 in the livers of SIV-infected macaques depicting portal regions (100X magnification, scale bar = 100 um). A no primary CCL2 antibody negative control was included to show specific staining (100X magnification, scale bar = 100 um). **F)** Immunohistochemical staining of recently infiltrated Mac387-positive monocyte/macrophages (brown cells) localized in the portal regions of the liver for uninfected, SIV+, and SIV+ ART macaques (100X magnification, scale bar = 100 um). **G)** Human monocyte-derived macrophages were stimulated with poly I:C or ssRNA40 at 0.2 and 2 ug/mL for 12 hours in duplicate. Relative expression of CCL2 was assessed by qRT-PCR comparing poly I:C and ssRNA40 stimulated cells to control (unstimulated) monocyte-derived macrophages. Data are graphed as the mean fold change in CCL2 expression ± SEM. Statistical significance was determined using a Mann Whitney T test comparing technical replicates of each condition to unstimulated cells with p ≤ 0.05 denoted as *.

Hepatic macrophage accumulation, and to a lesser extent infiltration of activated T cells, is largely driven by the CCL2-CCR2 chemokine/receptor axis, whereby CCL2 recruits CCR2-expressing monocytes/macrophages that differentiate into tissue macrophages with the potential to initiate macrophage-mediated inflammation [34–37][40]. Luminex assessment of plasma inflammatory factors revealed a significant increase in circulating levels of CCL2 in the blood of SIV-infected macaques compared to uninfected macaques (67 pg/mL in SIV-infected vs. 16 pg/mL in uninfected, p = 0.0158) (Fig 2A). To evaluate if CCL2 could be influencing T cell or monocyte/macrophage infiltration into the liver, the expression of CCL2 and its receptor, CCR2, were examined by qRT-PCR. We observed an intrahepatic upregulation of both CCL2 and CCR2 transcripts in SIV-infected untreated macaques (Fig 2B and 2C). Importantly, CCR2 expression levels correlate with hepatic CD68+ macrophage levels, providing evidence that CCL2/CCR2 axis contributes to the macrophage infiltration that was observed (Fig 2D). Although CCL2 levels did not directly correlate with macrophage levels, we did observe that in each treatment group those macaques with the highest CCR2 levels also tended to have high CCL2 expression (Fig 2B and 2C, individual macaques denoted with 1, 2, 3). To determine the location of the CCL2-producing cells within the liver, SIV-infected macaque livers were evaluated by immunohistochemistry. Diffuse CCL2 staining was observed throughout the liver with small foci of CCL2-producing cells in portal triad regions (Fig 2E).

To evaluate monocyte/macrophage recruitment in the liver during SIV infection, liver sections were evaluated for recently infiltrated monocytes/macrophages by staining for Mac387. The Mac387 protein recognizes the calcium-binding proteins MRP8 and MRP14 that are restricted to early stage monocyte differentiation and elevated in inflamed tissue, but are absent in mature tissue macrophages [41–43]. Additionally Mac387+ monocyte/macrophages potently migrate toward CCL2 gradients [44]. Elevated levels of Mac387-postive cells that were found localized around portal triads in the livers of SIV-infected macaques (Fig 2F). Furthermore, these Mac387+ monocyte/macrophages were markedly reduced in antiretroviral treated macaques (Fig 2F).

Upon pathogen sensing, many cell types in the liver have been implicated in the production of CCL2, including hepatocytes, hepatic stellate cells, sinusoidal endothelial cells and hepatic macrophages, with hepatic macrophages being the primary source of CCL2 [45]. Therefore, to mechanistically delineate the contribution of macrophages to CCL2 production during viral infection, monocyte-derived macrophages were stimulated with purified viral PAMPS, poly I:C, a TLR3 agonist, and ssRNA40, which is derived from the HIV-1 long terminal repeat and activates TLR7/8. These *in vitro* studies determined that viral PAMPs have the capacity to upregulate CCL2 expression in monocyte-derived macrophages with TLR7/8 activation using ssRNA40 producing higher CCL2 expression than poly I:C.

### Recruitment of CCR2-expressing T cells and inflammatory monocytes to the liver

**Fig 3:**
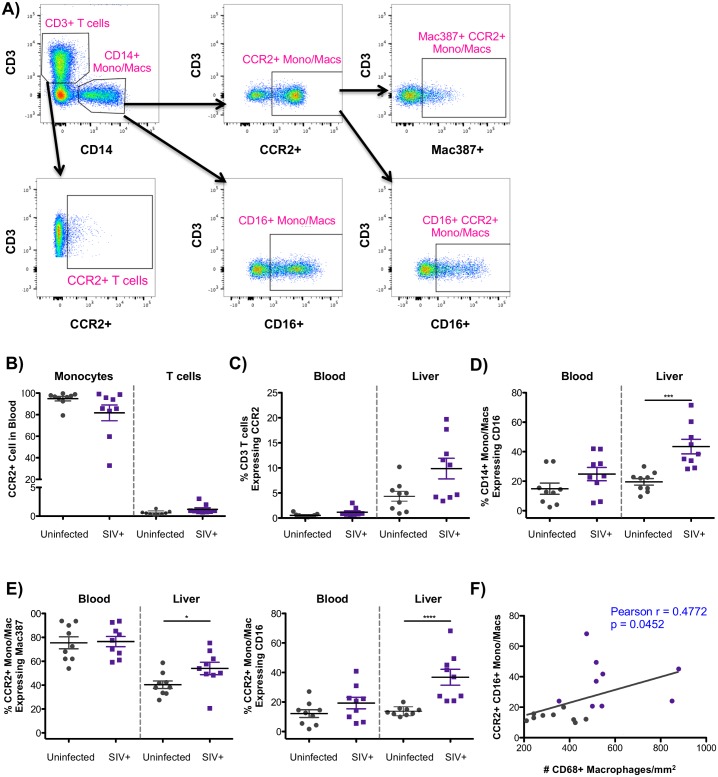
CCR2-associated influx of T cells and monocytes/macrophages in the liver during SIV infection. **A)** PBMC and liver cell suspensions were first gating on single, live, CD45+ cells followed by identification of T cells by CD3 expression and monocytes/macrophages by CD14 expression. T cells and monocytes/macrophages were each evaluated for CCR2 expression. Monocytes/macrophages were also assessed for CD16 expression, and the expression of Mac387 and CD16 on monocytes/macrophages expressing CCR2. **B)** Frequency of monocytes/macrophages and T cells expressing CCR2 in PBMC. **C)** Frequency of CD3+ T cells expressing CCR2 in the blood or in the liver. **D)** Levels of inflammatory CD16+CD14+ monocytes/macrophages in PBMC and in the liver. **E)** Phenotype of CCR2+ monocytes/macrophages in the blood and in the liver based on Mac387 and CD16 expression. **F)** Pearson Correlation evaluating the association between the levels of CCR2+CD16+ monocytes/macrophages in the liver (y-axis) and the number of CD68+ macrophages (x-axis) with uninfected macaques in gray and SIV-infected macaques in purple.

To characterize the influx of CCR2-expressing cells into the liver during infection, we assessed the phenotype of T cells and monocytes/macrophages in uninfected (n = 9) and SIV-infected (n = 9) macaques using flow cytometry. Both peripheral blood mononuclear cells (PBMC) and paired liver cell suspensions were first gated on singlets, live cells, and CD45 expression followed by CD3-positive T cells and the proportion of T cells expressing CCR2 (Fig 3A). Cells of monocyte/macrophage lineage were identified by CD14 expression followed by phenotyping using CD16 and CCR2. CCR2-expressing monocytes/macrophages were further characterized based on Mac387 and CD16 expression (Fig 3A). With regards to circulating PBMCs that have the capacity to migrate to CCL2 gradients in tissue, CCR2 was expressed on the majority of monocytes/macrophages while very few T cells expressed CCR2 in the blood (Fig 3B). However, when compared to blood, more T cells in the liver expressed CCR2 in both uninfected and SIV-infected macaques (Fig 3C) likely due to the fact that CCR2 is mostly expressed on activated T cells [46]. Additionally, five of the nine SIV-infected macaques displayed higher levels of CCR2-positive T cells in the liver, however this trend did not reach statistical significance (p = 0.077) (Fig 3C).

With regards to monocytes/macrophages, there was an observed expansion of inflammatory CD16+ monocytes/macrophages in the liver during SIV infection (Fig 3D). A similar trend for increased CD16+ monocytes/macrophages was detected in the blood, however this did not reach statistical significance (p = 0.1615). When considering CCR2+ monocyte/macrophages, there was an increase in the frequency of CCR2-positive monocyte/macrophages that recently infiltrated the liver based on expression of Mac387 (Fig 3E), providing evidence for active recruitment of monocyte/macrophages along the CCR2-CCL2 axis. In addition, there was a highly significant increase in CCR2-monocytes/macrophages that express the inflammatory marker, CD16, again providing evidence for CCR2-associated expansion of inflammatory CD16+ monocyte/macrophages in the livers of SIV-infected macaques. Moreover, these inflammatory monocytes/macrophages correlated with the number of CD68+ macrophages (Fig 3F).

**Fig 4:**
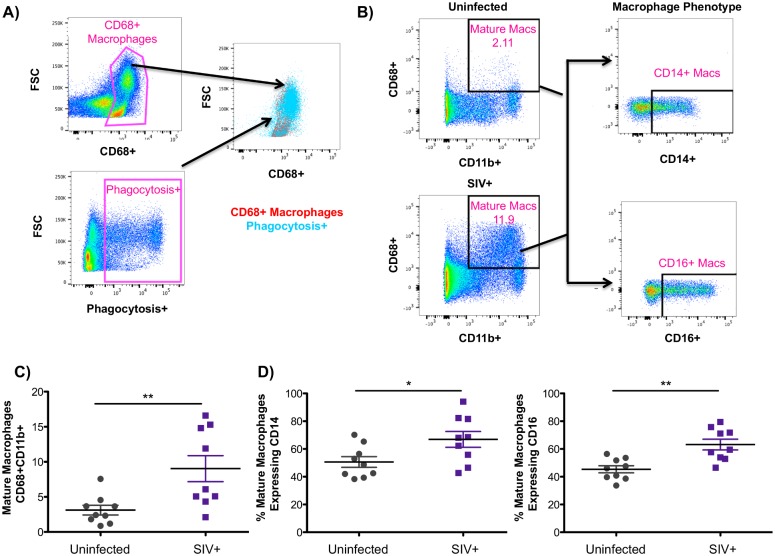
Evaluation of CD68+ mature macrophages in the liver. **A)** Freshly isolated macaque liver cells were incubated with fluorescently labeled E. coli bioparticles for 2 hours. After washing, cells were evaluated for phagocytosis of E.coli by flow cytometry. Gating first on single, live, CD45+, CD3- cells, CD68+ liver cells were found to be the primary cell subset to phagocytosis E. coli. **B)** Liver cells suspensions were first gated on single, live, CD45+, CD3- cells. Mature macrophages, identified as CD68+CD11b+, were evaluated for the expression of CD14 and CD16. **C)** Frequency of mature CD68+CD11b+ macrophages in the liver of uninfected (gray symbols) and SIV-infected (purple symbols) macaques. **D)** Phenotype of mature hepatic macrophages in uninfected (gray symbols) and SIV-infected (purple symbols) macaques based on CD14 and CD16 expression.

CD14 expression has been historically used to define cells of monocyte/macrophage lineage, however, tissue macrophages display considerable heterogeneity between different species and even in different tissue compartments. For example, CD14-negative tissue macrophages have been described in various tissues, including the liver, the gut, and the spleen [47, 48]. While the above analysis gated on CD14 to assess monocyte/macrophage populations in the liver (Fig 3), this gating strategy may not capture all mature tissue macrophages. CD68, on the other hand, is a well-characterized pan-macrophage marker that has been used to define tissue macrophages in macaques and in humans [49, 50]. Therefore, defining mature tissue macrophages as CD68+CD11b+, a previously defined phenotype of tissue macrophages in macaques [47], allowed us to interrogate the phenotype of mature hepatic macrophages during SIV infection. With regards to function, we observed that hepatic CD68+ cells were the predominant cell type involved in the phagocytosis of labeled E. coli, particularly those cells with higher forward scatter (Fig 4A). Gating first on single, live, CD45+, CD3- cells, mature CD68+CD11b+ macrophages were assessed for the expression of CD14 and CD16 (Fig 4B). Comparable to the assessment of CD68+ macrophages by immunofluorescence microscopy (Fig 1), our flow cytometry analysis also indicates a significant increase in the levels of mature macrophages in the liver during SIV infection (Fig 4C). Interestingly, our flow cytometry analyses revealed variable expression of CD14 on mature CD68+ macrophages with SIV-infected macaques having more CD14+CD68+ macrophages than uninfected macaques (66.9% vs 50.6% of macrophages expressing CD14). A similar trend was observed regarding CD16 expression during infection (63.3% vs 45.4% of macrophages expressing CD16) indicating that these mature macrophages display an altered phenotype during SIV infection with a higher percentage expressing CD14 and CD16 (Fig 4D).

### Levels of bacterial 16S DNA in the liver during SIV infection

**Fig 5:**
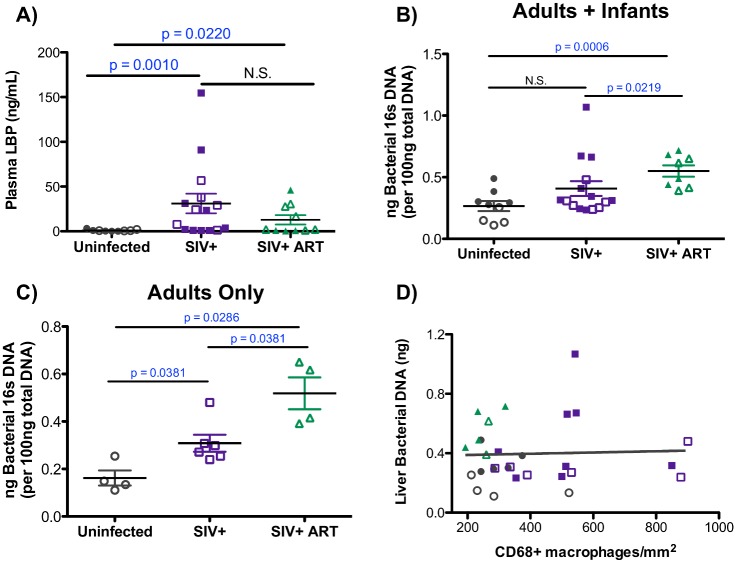
Levels of microbial products in the liver and in the plasma during SIV infection. **A)** Plasma LBP levels were quantified in duplicate for each macaque by ELISA. Data are graphed as the mean LBP concentration (ng/mL) for each macaque ± SEM. Statistical significance was determined by Mann Whitney T tests. **B-C)** Levels of bacterial 16S DNA was quantified by qPCR for each liver sample assayed in duplicate. Nanograms (ng) of bacterial DNA present in 100 ng of total input DNA was determined using a standard curve and graphed as the mean of each macaque assayed in duplicate ± SEM. Infant (closed symbols) and adult macaques (open symbols) graphed together (B) or adult macaques graphed separately (C). Statistical significance was determined by Mann Whitney T tests. **D)** Correlation between the levels of 16S DNA in the liver (y-axis) and the number of liver macrophages (x-axis) in uninfected (gray points), SIV-infected (purple symbols), and SIV-infected cART macaques (green symbols) as evaluated by Spearman Correlation analysis.

The detection of microbial products within the blood during HIV and SIV infections is well established [31, 32, 51]. To determine if elevated levels of liver macrophages were associated with systemic bacterial translocation, the concentration of plasma LPS-binding protein (LBP) was assessed. A significant increase was observed in circulating levels of LBP in SIV-infected as well as in SIV-infected cART-treated macaques (Fig 5A). The presence of elevated LBP levels suggests that more bacteria are translocating through the liver, as the liver plays an important physiological role in the detoxification of microbial products that enter via the portal vein. Therefore, liver bacteria levels were quantified using the 16S rRNA gene by qPCR. Bacterial DNA was detected in all of the liver samples, indicating that uninfected macaques normally have detectable levels of bacterial DNA (Fig 5B). Interestingly, uninfected infant macaques were found to have elevated levels of bacterial DNA within their livers compared to uninfected adults (0.35 ng vs 0.16 ng 16S DNA/100ng total DNA), a difference that was statistically significant (p = 0.0159) (Fig 5B). Assessing the adult and infant macaques together determined that SIV-infected cART-treated macaques had significantly elevated levels of liver 16S DNA when compared to uninfected (p = 0.0006) and SIV-infected macaques (p = 0.0219) (Fig 5B). The lack of a significant difference in the levels of 16S DNA within SIV-infected macaques when compared to uninfected macaques was due to the higher levels of 16S DNA in the uninfected infants (Fig 5B), as significantly elevated levels of liver 16S DNA was observed when adult macaques were evaluated alone (Fig 5C). The elevation in liver 16S DNA levels in the SIV-infected cART-treated macaques was unexpected, but nevertheless, this elevation of bacterial DNA in the livers of the SIV-infected and SIV-infected cART-treated macaques did not correlate with macrophage levels (Fig 5D), indicating that the macrophage expansion observed during infection is likely not mediated by bacterial translocation to the liver.

### Liver macrophage and T cell infiltration are associated with SIV burden

**Fig 6:**
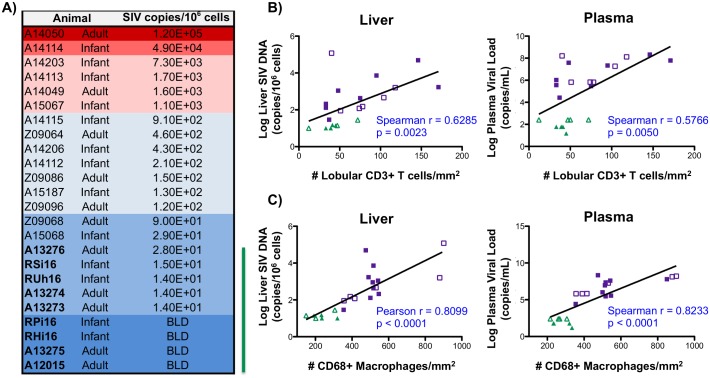
Liver macrophage number correlates with SIV levels. **A)** SIV DNA copies in samples from the livers of both infant and adult macaques were determined by quantitative hybrid real-time/digital PCR and normalized per million cell equivalents, based on parallel analysis of a single copy rhesus macaque CCR5 target template. Macaques treated with cART are denoted with bolded font and a green line to the right of the table (BLD = below the limit of detection). **B-C)** Correlation analysis between copies of SIV DNA in the liver (left) or plasma SIV levels (right) and liver T cell number (B) or liver macrophage number (C) in SIV-infected (purple symbols) and SIV-infected cART (green symbols) macaques. For macaques having SIV plasma viral load and SIV DNA copies below the limit of detection, the log transformed limit of detection values (30 copies/mL for plasma or 10 copies/10^6^ cells for liver) were used for correlation analysis. Adult (open symbols) and infant (closed symbols) macaques are denoted.

To assess the levels of SIV DNA in the liver and to provide insights regarding the role of virus in driving macrophage infiltration, SIV DNA was quantified by quantitative hybrid real-time/digital PCR [52, 53]. We observed a wide range of SIV levels (per 10^6^ cell equivalents) in the livers of SIV-infected macaques with no distinct differences associated with age (Fig 6A). As expected, macaques treated with cART had the lowest levels of SIV with four of the macaques having levels below the limit of detection for the assay (<10 SIV DNA/10^6^ cell equivalents) (Fig 6A). A correlation was observed between the level of SIV DNA in the liver and plasma SIV RNA and the number of hepatic T cells, which were quantified by immunofluorescence microscopy (Fig 1)(Fig 6B). Further analysis identified an even stronger correlation between the levels of SIV DNA hepatic macrophage levels (r = 0.8099, p<0.0001) (Fig 6C). This correlation with liver macrophage number was also observed when plasma SIV RNA levels were assessed (p<0.0001, Fig 6C). These findings demonstrate a direct relationship between SIV load in liver and the levels of hepatic T cells and macrophages implicating SIV burden as a key stimulus in recruitment of immune cells into the liver.

### SIV-positive cells are predominately T cells that localize in portal areas

**Fig 7:**
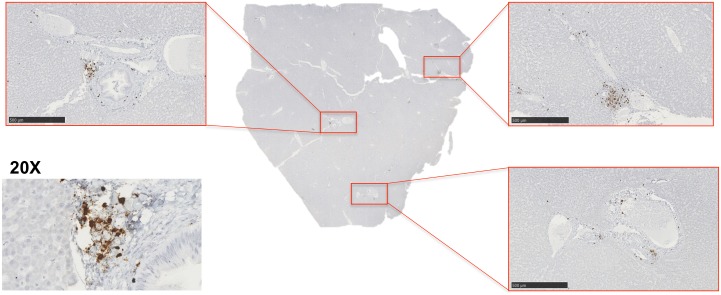
SIV-infected cells localize around the portal triads. **A)** RNAscope in situ hybridization was used to detect SIV RNA-positive cells in the liver. Images were captured from whole tissue scans at 5x magnification (images outlined in red, scale bar = 500 um) and depict SIV-infected cells around the portal triads. A representative 20x image of a portal triad was included of the SIV-RNA positive cells (brown cells).

**Fig 8:**
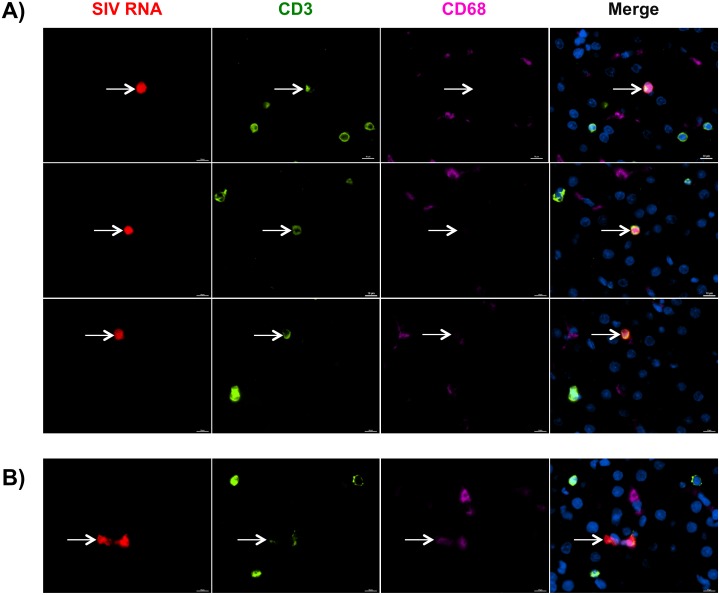
T cells are the primary cellular subset infected with SIV in the liver. **A-B)** Liver tissue sections from SIV-infected untreated macaques were assessed for SIV RNA+ cells (red) by *in situ* RNAscope technology followed by antibody staining with CD3 to identify T cells (green) and CD68 to identify macrophages (pink). Nuclei were stained with Dapi (blue). SIV RNA+ signal was predominately found associated with CD3 T cells (A) and in rare cases CD68 macrophages (B).

Structurally composed of lobules, the liver is a multifaceted organ with distinct tissue structures that represent unique environments for SIV replication. The high levels of SIV DNA in the liver and the observed correlations with both macrophages and T cells raised questions with regard to where in the liver SIV replication was occurring and which cells, macrophages or T cells, were producing virus. Therefore, utilizing highly sensitive RNAscope *in situ* hybridization technology [21, 54, 55], we assessed the location and phenotype of SIV RNA-positive cells in the livers of SIV-infected untreated macaques. We identified SIV RNA-positive cells predominately localized to the portal triad regions of the liver with lower levels of SIV-positive cells located in the lobular tissue (Fig 7). SIV RNA-positive cells that were found in lobular regions were typically a single, individual cell, not large aggregates of SIV-positive cells like those observed in the portal regions. To identify the cellular subset associated with SIV replication in the liver, RNAscope was used in combination with antibody staining for CD3 and CD68 to identify T cells and macrophages, respectively. We observed that most SIV RNA was associated with T cells (Fig 8A). Occasionally, SIV RNA-positive signal co-localized with CD68+ macrophages (Fig 8B), however, this was quite rare and often there was diffuse CD3 staining in the same area suggesting this may be a macrophage that has acquired SIV RNA by T cell phagocytosis as has been demonstrated in previous studies [56]. Although SIV DNA was detected in the liver during cART, we were unable to detect SIV RNA-positive cells in the livers of macaques treated with antiretroviral drugs by RNAscope.

### Liver macrophages correlate with increased levels of immune mediators associated with inflammation

**Fig 9:**
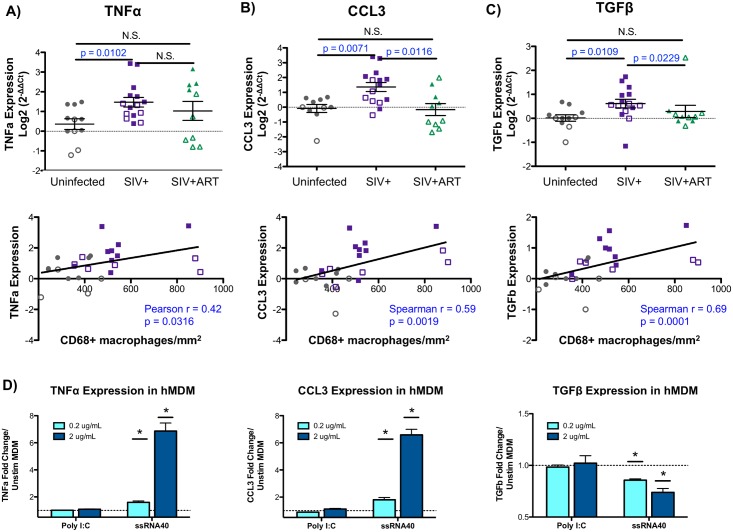
Liver macrophage number correlates with inflammatory and fibrosis mediators. **A-C)** Quantitative PCR was used to evaluate the hepatic expression of macrophage-associated chemokines and cytokines using the comparative threshold method. Data are graphed as the Log2 transformed mean ± SEM with adult (open symbols) and infant (closed symbols) macaques are denoted in each graph. Statistical significance was determined by Mann Whitney T tests. Correlation analysis was used to evaluate the relationship of cytokine/chemokine expression with liver macrophage number. Data displaying normal distribution were analyzed by Pearson correlation with data not passing normality were assessed by Spearman correlation. A significant increase in TNFα **(A)**, CCL3 **(B)**, and TGFβ **(C)** was observed in livers of SIV-infected macaques that positively correlated with liver macrophage number in the uninfected (gray points) and SIV-infected (purple points) macaques. **D)** Human monocyte-derived macrophages were stimulated with poly I:C or ssRNA40 at 0.2 and 2 ug/mL for 12 hours in duplicate. Relative expression of TNFα, CCL3 and TGFβ was assessed by qRT-PCR comparing poly I:C and ssRNA40 stimulated cells to control (unstimulated) monocyte-derived macrophages. Data are graphed as the mean fold change in expression ± SEM. Statistical significance was determined using a Mann Whitney T tests comparing technical replicates of each condition to unstimulated cells with p ≤ 0.05 denoted as *.

To gain further insights into the mechanisms of SIV-induced liver inflammation, the hepatic expression of pro-inflammatory (CCL3, TNFα), pro-fibrosis (TGFβ), and anti-inflammatory (IL-10) mediators were evaluated by qRT-PCR. There was a significant increase in both CCL3 and TNFα in the livers of SIV-infected macaques when compared to uninfected animals (Fig 9A and 9B). A similar upregulation was observed in the pro-fibrosis cytokine, TGFβ, in macaques that were SIV-infected (Fig 9C). Following cART, both CCL3 and TGFβ were significantly reduced in the liver while TNFα remained elevated, particularly in infant macaques (Fig 9A, 9B and 9C). In contrast, assessment of the inflammation-suppressive cytokine, IL-10, showed no differences between treatment groups when both adults and infants were evaluated together (S2 Fig). Interestingly, infant macaques had higher expression of IL-10 transcripts in the liver (S2 Fig) and increased circulating levels of IL-10 in the blood (S2 Fig) when compared to adults, likely reflecting inherent differences in immune system maturity between the age groups.

Correlation analyses demonstrated a significant, positive correlation between hepatic macrophage levels and the expression of liver inflammatory mediators (TNFα, CCL3), consistent with a role for liver macrophages as inflammatory contributors (Fig 9A and 9B, lower panels). Similarly, TGFβ levels also correlated with hepatic macrophage number suggesting that infiltrating macrophages in the SIV-infected macaques may trigger the early events that may lead to liver pro-fibrosis responses (Fig 9C, lower panel).

To delineate the role that monocyte-derived macrophages may have in driving the expression of inflammatory and pro-fibrosis mediators during infection, *in vitro* generated human monocyte-derived macrophages were stimulated with viral-associated PAMPs. We observed that monocyte-derived macrophages were able to upregulate both inflammatory mediators, CCL3 and TNFα (Fig 9D). This upregulation was observed with ssRNA40, which is derived from HIV and activates a main pathogen-recognition receptor for HIV, TLR7/8. Interestingly, TGFβ was significantly downregulated by viral PAMP stimulation using ssRNA40 (Fig 9D) suggesting that macrophages that infiltrate the liver during infection may not be the producers of TGFβ.

### Liver transcriptome changes in SIV-infected and SIV-infected cART-treated macaques

**Fig 10:**
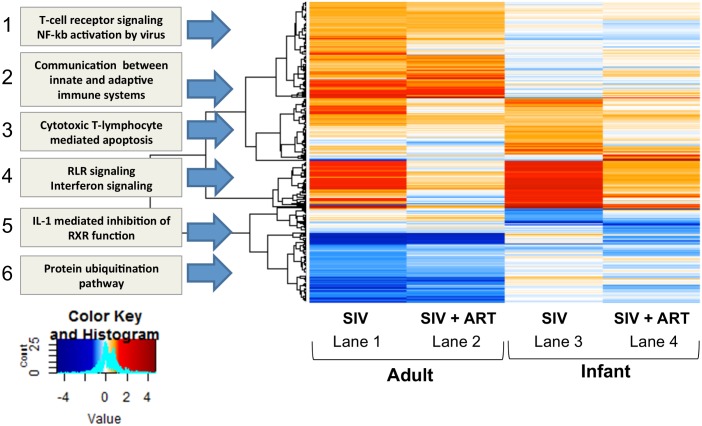
Heatmap of the global liver transcriptomic signature. The liver transcriptome was evaluated by microarray analysis. Treatment groups (SIV+ or SIV+ cART) were compared against age-matched uninfected macaques to determine differentially expressed genes using the limma package in R/bioconductor. Co-expression analysis was performed using weighted gene coexpression network analysis (WGCNA) and heatmap packages in R/bioconductor using cutoffs of 1.5 fold change and 0.05 adjusted pvalues in at least one condition. Gene networks (modules) involved in immune response and inflammation are upregulated in SIV-infected macaques with antiviral RLR and interferon signaling highly upregulated in both adults and infants.

To elucidate the global impact of SIV infection and cART treatment on overall liver homeostasis, the hepatic transcriptomic signature was characterized during chronic SIV infection by microarray expression analysis. Differentially expressed genes were determined by normalizing SIV-infected and SIV-infected cART macaques to age-matched uninfected controls. Overall, liver transcriptional changes in SIV-infected macaques included a number of different functional pathways with the most significant changes occurring in immune and inflammation-associated genes, which are depicted in the functional modules outlined in Fig 10. The SIV-infected adult macaques exhibited an increase in the first four modules, and a down- modulation in the last two pathways as indicted in the heatmap (Fig 10, lane 1). Some of the interesting observations are the upregulation of T cell signaling and NF-kb activation (module 1) and genes associated with communication between innate and adaptive immune systems (module 2), both of which continue to be upregulated, although to a lesser extent, in macaques that were administered cART (Fig 10, lane 2). A strong upregulation in Rig-I-like receptor (RLR) signaling/interferon response (module 4) was also evident in the untreated SIV-infected adult macaques with many of the top upregulated genes being involved in antiviral defense (e.g. ISG15, MX1) (Table 1). Finally, an interesting down-regulation was observed in IL-1 mediated inhibition of retinoid X receptor (RXR) function (module 5) as RXR activity plays a role in metabolic disorders and in the regulation of several macrophage functions, including production of chemokines, pathogen sensing, and macrophage lipid metabolism [57].

**Table 1:** Top differentially expressed genes in SIV+ adult macaques.

Gene	Log2 F.C.	Adjusted p-value	Gene Ontology
ISG15	3.798	0.0383	interferon response, defense response to virus
MX1	3.651	0.0263	type I interferon signaling, innate immune response, response to virus
IFI6	3.545	0.0107	type I interferon signaling, immune response
IFIT1	3.050	0.0484	type I interferon signaling, response to virus
HERC6	2.906	0.0250	protein polyubiquitination
PPM1K	2.524	0.0075	branched-chain amino acid catabolism, protein dephosphorylation
ALAS1	2.522	0.0057	regulation of lipid metabolism, heme biosynthesis, mitochondrion organization
IFIT3	2.417	0.0297	type I interferon signaling, response to virus
FN1	2.287	0.0057	acute-phase response, extracellular matrix organization, regulation of fibroblast proliferation
OAS2	2.165	0.0372	type I interferon signaling, response to virus
EPSTI1	2.103	0.0381	nuclear translocation of NF-kB
OAS3	2.058	0.0402	type I interferon signaling, response to virus
UGT1A1	1.926	0.0246	acute-phase response, bilirubin conjugation, cellular glucuronidation, liver development
EIF2AK2	1.913	0.0172	innate immune response, response to virus, regulation of NF-kB transcription factor activity
NR0B2	1.873	0.0498	organ regeneration, cholesterol metabolism, response to glucose, regulation of insulin secretion
TEX264	1.828	0.0031	platelet degranulation
PER1	1.783	0.0138	circadian rhythm, regulation of glucocorticoid receptor signaling, regulation of cytokine production
GLYAT	1.762	0.0376	glycine metabolism, monocarboxylic acid metabolism, response to toxic substance
SNAI3	1.751	0.0376	regulation of transcription from RNA polymerase II promoter
HLA-B	1.724	0.0081	antigen processing and presentation via MHC class regulation of immune response

In contrast to adults, SIV infection of infant macaques resulted in an upregulation of only two pathways, cytotoxic T lymphocyte-mediated apoptosis (module 3) and RLR/interferon signaling (module 4) (Fig 10, lane 3). Many of the antiviral RLR/interferon genes that were found upregulated in adults were also upregulated in SIV-infected infant macaques (bolded genes, Table 2). Since the RLR signaling/interferon signature was found in both the adults and infants during SIV infection, the specific genes involved in this pathway were examined by pathway analysis. During viral infections, such as SIV, genes in the RLR pathway code for proteins that are involved in sensing cytosolic RNA viruses and/or viral PAMPs, the production of interferons (e.g. IFNα/β) and the downstream activation of interferon-stimulated genes (ISGs) [58]. The pathway analysis revealed the expression of several interferon-induced signaling molecules (e.g. Stat1, IRF9) and downstream interferon stimulated genes (ISGs) (e.g. Mx1, OAS1) was altered in SIV-infected infants (S3 Fig). This was further confirmed through network analysis where many antiviral response genes were identified to be strongly upregulated in the liver, including IRF7, IRF9, ISG15, and Mx2 (S3 Fig). Interestingly, CCL3, one of the pro-inflammatory mediators assessed by qRT-PCR that correlated with liver macrophage number, was also identified in this network analysis and predicted to be part of the host antiviral response (S3 Fig). The pathway analyses for SIV-infected adult macaques, which contained slightly more variation, identified similar gene activity, but fewer genes met the cut-off criteria (p < 0.05, fold change >1.5) (S4 Fig) compared to the infant macaques.

**Table 2:** Top differentially expressed genes in SIV+ infant macaques.

Gene	Log2 F.C.	Adj p-value	Gene Ontology
HRASLS2	4.227	0.0368	lipid catabolism, phosphatidylethanolamine acyl-chain remodeling
**ISG15**	3.676	0.0082	type I interferon signaling, response to virus
**IFI6**	3.496	0.0002	type I interferon signaling, immune response
IFIT1B	3.492	0.0197	defense response to virus
**MX1**	3.458	0.0042	type I interferon signaling, response to virus, innate immune response
Saa3	3.290	0.0400	acute-phase response, I-kappaB phosphorylation, cell chemotaxis
OAS1	3.266	0.0031	type I interferon signaling, response to virus, glucose homeostasis
**IFIT1**	3.163	0.0079	type I interferon signaling, response to virus
SAA1	3.031	0.0370	acute-phase response, innate immune activation, macrophage chemotaxis
OASL	2.900	0.0021	type I interferon signaling, response to virus
**HERC6**	2.629	0.0059	protein polyubiquitination
**IFIT3**	2.559	0.0016	type I interferon signaling, response to virus
CMPK2	2.486	0.0048	cellular response to LPS, terminal differentiation of monocytic cells
IRF7	2.417	0.0246	regulation of type I interferon production, innate immune response, defense response to virus
**EPSTI1**	2.403	0.0014	nuclear translocation of NF-kappaB
**OAS2**	2.332	0.0025	type I interferon signaling, response to virus
H2-M2	2.308	0.00002	antigen processing and presentation via MHC class I
LBP	2.264	0.0033	acute-phase response, cellular response to LPS and lipoteichoic acid, macrophage activation
HLA-G	2.261	0.000007	antigen processing and presentation via MHC class I, regulation of tolerance induction
MX2	2.229	0.0043	type I interferon signaling, response to virus

In SIV-infected macaques receiving cART, the liver antiviral signature was reduced, but not completely, especially in infant macaques (Fig 10, module 4, lanes 2 and 4). It could be that residual virus or elevated bacteria levels, which were detected in the liver during cART, may drive some persistent liver inflammation during treatment. As the antiviral profile decreased during cART, we observed that many of the top upregulated genes in both adult and infant macaques are involved in metabolism, cellular homeostasis and the oxidation-reduction process (e.g. SLC22A4, Akr1b7) (Tables 3 and 4). Collectively, this suggests the liver continues to experience perturbation of function during drug therapy, and could explain some of the fatty liver complications observed in HIV-infected individuals.

**Table 3:** Top differentially expressed genes in SIV+ ART adult macaques.

Gene	Log2 F.C.	Adj p-value	Gene Ontology
RABIF	2.188	0.0222	protein transport, regulation of GTPase activity, membrane fusion
SH3KBP1	1.958	0.0494	apoptosis, cytoskeleton organization, cell migration
SLC22A4	1.572	0.0347	organic anion and cation transport, triglyceride metabolism, body fluid secretion
Cmah	1.565	0.0390	CMP-N-acetylneuraminate metabolism, oxidation-reduction process
CYP2F1	1.480	0.0164	xenobiotic metabolism, response to toxic substance, oxidation-reduction process
SLC2A4RG	1.446	0.0280	regulation of transcription from RNA polymerase II promoter
SECISBP2L	1.416	0.0018	selenocysteine incorporation
SYTL2	1.400	0.0347	exocytosis, intracellular protein transport, protein localization to plasma membrane
PAQR7	1.296	0.0306	response to steroid hormone, steroid hormone mediated signaling
AUTS2	1.256	0.0385	regulation of transcription from RNA polymerase II promoter
ZNF704	1.235	0.0222	regulation of transcription from RNA polymerase II promoter
FZD4	1.189	0.0359	Wnt signaling pathway, extracellular matrix-cell signaling, ubiquitin protein ligase binding
HFE2	1.152	0.0385	BMP signaling, cellular iron ion homeostasis
MTMR1	1.107	0.0347	phosphatidylinositol biosynthesis and dephosphorylation
EGFL7	1.099	0.0347	Notch signaling, cell adhesion, regulation of endothelial cell proliferation
GPC4	1.077	0.0280	Wnt signaling, cell proliferation, glycosaminoglycan metabolism
NPTX1	1.072	0.0339	chemical synaptic transmission
ROBO2	1.068	0.0280	cellular response to hormone stimulus, regulation of negative chemotaxis
PDCD6IP	1.043	0.0496	apoptosis, bicellular tight junction assembly, maintenance of epithelial cell apical/basal polarity
STXBP5	1.024	0.0397	exocytosis, protein transport, regulation of protein secretion

**Table 4:** Top differentially expressed genes in SIV+ ART infant macaques.

Gene	Log2 F.C.	Adj p-value	Gene Ontology
Akr1b7	3.687	0.0358	cellular lipid metabolism, oxidation-reduction process,
AKR1B10	3.671	0.0243	farnesol catabolism, oxidation-reduction process, retinoid metabolism
SREBF1	2.298	0.0221	cellular response to fatty acid, cholesterol metabolism, insulin signaling, lipid metabolism
UCHL1	2.205	0.0007	cell proliferation, protein deubiquitination
NQO1	1.777	0.0333	removal of superoxide free radicals, response to toxic substances, xenobiotic metabolism
ACSS2	1.597	*0*.*0651*	lipid biosynthesis, mitochondrion organization
CTSE	1.466	0.0028	antigen processing and presentation via MHC class II, protein catabolism
STARD4	1.331	0.0366	cholesterol import/intracellular transport, regulation of bile acid biosynthesis
SEMA4D	1.052	0.0221	cell adhesion, immune response, regulation of cell migration, regulation of leukocyte aggregation
NANOS2	1.003	0.0109	cell differentiation, mRNA catabolism
PRDX1	1.000	0.0480	cellular response to oxidative stress, hydrogen peroxide catabolism
NR1D2	1.000	*0*.*0904*	lipid homeostasis, regulation of lipid metabolism, energy homeostasis
VAX2	0.944	0.0074	Wnt signaling, regulation of transcription from RNA polymerase II promoter
SLC9A3R1	0.869	0.0368	Wnt signaling, bile acid secretion, regulation of cell proliferation and migration
PAFAH1B3	0.850	0.0221	lipid metabolism
EMC9	0.848	0.0366	protein binding
SENP2	0.793	0.0096	Wnt signaling, protein destabilization, regulation of protein ubiquitination
ABHD4	0.765	0.0232	lipid catabolism, phophatidylethanolamine acyl-chain remodeling
NPC1L1	0.760	0.0109	response to drug, cholesterol biosynthesis, intestinal cholesterol and lipid absorption
BLVRB	0.714	0.0232	heme catabolism, oxidation-reduction process

## Discussion

Liver disease has emerged as one of the most common non-AIDS-related causes of death in those infected with HIV, accounting for 14%-18% of all deaths [59]. Here, a pathogenic SIV-macaque model was utilized to provide insights into liver disease pathogenesis during SIV infection and the impact of antiretroviral treatment. Our findings identify a correlation between the levels of CCL2-CCR2 and immune cell trafficking to the liver during SIV infection, including monocytes/macrophages and T cells. Infiltration of these immune cells during untreated SIV infection is likely driven by SIV burden as correlations between immune cell number, both macrophages and T cells, and SIV levels were observed, in addition to a strong antiviral transcriptiomic signature detected in the microarray analyses. These findings provide mechanistic insights into how infiltration of monocytes/macrophages into the liver can induce tissue inflammation during SIV infection and also suggest that viral suppression during cART can greatly reduce SIV-associated liver inflammation, although these levels are still elevated compared to uninfected macaques.

Expansion of the liver macrophage population has been implicated in multiple forms of liver disease, including acute liver failure, HCV/HBV infection, NAFLD, fibrosis, and alcoholic liver disease [40, 60]. Moreover, studies elucidating the role of monocytes/macrophages in SIV pathogenesis have indicated that monocyte/macrophage accumulation correlates with SIV disease severity and progression, tissue damage in the lung and gut, and SIV-associated encephalitis [61–63]. Importantly, inhibiting macrophage accumulation has shown therapeutic benefits in models of both liver disease and SIV infection [36, 38, 39, 64, 65]. In our study, we observed an increase in hepatic monocyte/macrophages during SIV infection that correlated with both inflammatory (TNFα, CCL3) and fibrosis (TGFβ) mediators suggesting multiple, and maybe even opposing, roles during infection. Interestingly, our experiments utilizing monocyte-derived macrophages, reveals that these monocyte-derived macrophages are potent producers of inflammatory mediators, both TNFα and CCL3, but not TGFβ, at least in the *in vitro* environment. Therefore, we hypothesize the production of TGFβ *in vivo* may represent a compensatory mechanism to limit liver inflammation induced by infiltrating monocytes/macrophages and that other cell types in the liver may also contribute to TGFβ production, including sinusoidal endothelial cells, resident Kupffer cells, intrahepatic lymphocytes and dendritic cells [66]. It was interesting that TGFβ transcript levels decreased in the macaques that were treated with antiretroviral drugs when compared to untreated SIV-infected macaques given that some human studies report progression of liver fibrosis during cART. However, many of these studies found liver fibrosis to be associated with other factors during drug therapy, including poor viral control, low CD4 T cell counts, presence of HCV infection, older age, and alcohol abuse [10, 12, 67, 68], which were not recapitulated in our SIV-macaque experiments.

The accumulation of diverse monocyte/macrophage subsets in different tissue compartments during HIV and SIV infection has been described [27, 61–63, 69–72]. For example, CD163+ macrophages, a noninflammatory subset, accumulate in the gut while inflammatory Mac387+ macrophages infiltrate the brain [61, 63] suggesting disparate roles for macrophages during infection. Monocyte/macrophage accumulation in the liver was characterized by an expansion of both inflammatory CD16+CD14+ monocyte/macrophages and mature CD68+ macrophages during SIV infection. CD16+ monocytes/macrophages are presumed to represent a more activated phenotype and are elevated in the periphery in a number of chronic inflammatory conditions, including arthritis, atherosclerosis, Crohn’s disease and even during HIV/SIV infection [73–75]. In the context of liver disease, hepatic CD16+ monocytes/macrophages are potent producers of reactive oxygen species and secrete high levels of inflammatory chemokines and cytokines [76] with the highest levels of these cells found in patients with liver cirrhosis [77]. Although we cannot definitively determine if these inflammatory CD16+ monocyte/macrophages differentiate into tissue macrophages using our current model, Sugimoto et al. utilized BrdU labeling in rhesus macaques to demonstrate the capacity of CD14+CD16- classical monocytes to gradually progress to CD14+CD16+ monocytes and that these monocytes may differentiate into tissue macrophages [78]. Taken together, this suggests that the accumulation of these inflammatory CD16+ monocytes/macrophages is likely a key mediator of liver inflammation and subsequent hepatic dysfunction during SIV infection.

In both humans and rhesus macaques, replenishment and/or expansion of tissue macrophages due to infection or injury is most often associated with monocyte egress from bone marrow and entrance into systemic circulation in the blood, followed by homing to tissue and differentiation into mature tissue macrophages [79]. To further characterize the accumulation of hepatic monocytes/macrophages during SIV infection, we evaluated the CCL2-CCR2 chemokine network, a key inducer of monocyte/macrophage infiltration into the liver. We observed an upregulation of both CCL2 and CCR2 in the liver in macaques during untreated SIV infection with CCR2 expression positively correlating with CD68+ macrophages. Our flow cytometry evaluation of liver cells implicates CCR2 in the active recruitment of immune cells to the liver, where we observed elevated levels of CCR2+ T cells and CCR2+ monocyte/macrophage subsets. Our results suggest that SIV virus, not bacteria, induces immune cell trafficking to the liver as SIV levels in the liver and plasma correlated with both hepatic T cell and macrophage numbers, but bacteria levels did not. In fact, elevation of CCL2 has been described in multiple compartments (blood, gastronintestinal tract, the brain) during HIV infection with CCL2 levels correlating with viremia [80–82]. Moreover, HIV is a potent inducer of CCL2 production from many cell types *in vitro*, including liver stellate cells [83–85]. Therefore, we speculate that viral stimulation in the liver alters the immune environment through induction of CCL2, and possibly other chemokines, resulting in immune cell infiltration.

Another chemokine, inflammatory CCL3, which also correlated with macrophage number, is of major interest due to its ability to attract CCR5-expressing HIV/SIV target cells to the liver and the fact that this chemokine is involved in the retention of T cells at portal triads [86], which is where we observe SIV-infected cells to be primarily located in the liver. Previous studies have identified macrophages as key producers of CCL3 during viral infection and that CCL3 production is dependent on host interferon signaling [87]. Regarding its role during HIV infection, CCL3 is produced by macrophages following HIV exposure *in vitro* [88, 89], is upregulated in the livers of HIV-HCV co-infected individuals [90], and is linked to reduced CD4 T cell counts [91]. Collectively, this implies that under viral stimulation, macrophage production of CCL3 may directly influence infiltration of SIV-target cells, mainly T cells, to further amplify SIV levels and inflammation.

The portal region was particularly impacted during infection characterized by massive infiltration of immune cells, production of CCL2, and high levels of SIV-RNA positive cells. During some forms of chronic liver inflammation, portal tracts can organize into lymphoid follicles to form a specialized microenvironment for the recruitment and retention of activated immune cells in the chronically inflamed liver [92]. The factors that determine distribution of immune cells within the liver are poorly understood, but are likely controlled by differential expression of chemokines and adhesion molecules between the portal tract and the parenchyma. For example, homing to the portal triad involves RANTES, CCL2, and CCL3 while IP-10, MIG and ITAC have been implicated in recruitment to the sinusoids [93]. The immune signature captured in this study involving the upregulation of the chemokines CCL2 and CCL3, in combination with the various immune cell subsets aggregating at portal triads is consistent with portal disease during SIV infection.

We observed that T cells were the predominant cell type positive for SIV-RNA in the liver, which is consistent with other tissue compartments during normally progressing HIV and SIV infection [21, 94]. Regarding infection of hepatic macrophages, SIV RNA-positive macrophages were sparsely located throughout the lobular tissue of infected macaques. However, questions still remain as to whether these are truly productively infected macrophages or macrophages engulfing infected CD4 T cells. Several models of SIV infection have demonstrated infection of macrophages in several tissue compartments, including the spleen, lung, and brain, which is generally associated with a loss of CD4 T cells and accelerated disease progression [95–97]. Experimental depletion of CD4 T cells in macaques results in expansion of SIV-infected macrophages and microglia [98] demonstrating macrophage infection increases during disease progression as CD4 T cells decline. With regards to the liver, hepatic macrophages do not support viral replication *in vivo* [99] and also express low levels of CD4 and co-receptors, CCR5/CXCR4 [27], suggesting they may not be permissive to infection. Therefore, given the low number of SIV RNA-positive macrophages observed in our study, we speculate that macrophage infection occurs at low levels during normal disease progression in the liver.

Regarding the impact of antiretroviral drug intervention on liver inflammation, we observed a loss of macrophage accumulation, a reduced antiviral transcriptomic profile and decreased inflammation, which coincided with reduced levels of SIV DNA. SIV DNA was detected in the liver above the limit of detection for three of the five cART adult macaques analyzed, which has also been reported in human HIV infection during drug therapy [100]. Residual virus in the liver may be attributed to the role that the liver plays in clearance of activated T cells and SIV from circulation [26, 101]. Notably, although viral levels decreased in the liver during therapy, bacteria levels actually increased suggesting that gut barrier dysfunction persisted during cART. Murine studies have demonstrated elevated levels of bacteria compartmentalized in the liver during intestinal inflammation, and in models of experimentally induced liver disease, profound defects in bacterial clearance from the blood and heightened immune activation occur [102]. Therefore, although microbial translocation persists from the gut during antiretroviral therapy, liver function may improve, allowing for better clearance of microbial products. Indeed, we observed that LBP levels, a marker of bacterial translocation, decreased in the plasma during cART, but that liver bacteria levels increased suggesting the liver is filtering more microbial products from portal vein and systemic circulation.

Although the impact of elevated liver microbes in the context of SIV-associated liver dysfunction will warrant additional studies, gut microbial translocation is recognized as a possible cause of fatty liver disease and other metabolic syndrome manifestations, involving increased lipid peroxidation, ROS, and systemic inflammation [103]. Here, in macaques treated with antiretroviral drugs, there was observed alteration in metabolism characterized by downregulation in genes associated with ‘IL-1 mediated inhibition of RXR function’ in the transcriptome. In the context of liver function, perturbation of the retinoid X receptor (RXR) pathway is significant as RXR is a nuclear receptor that regulates transcription of many enzymes involved in the metabolism of lipids, cholesterol, bile acids, and xenobiotics (e.g. cART drugs). Altered activity of RXR with its heterodimers (e.g. PPAR, FXR, LXR) enhances fatty acid synthesis and/or alters fatty acid hepatic export leading to the accumulation of lipids in the liver (steatosis) [104], a common complication of HIV-infected people. Many of the top differentially expressed genes in antiretroviral-treated macaques are involved in metabolism suggesting that metabolism imbalance is prominent in these macaques.

There is limited understanding of how HIV infection impacts liver function in the absence of other factors that confound human studies, such as viral hepatitis co-infection, alcohol use, injection drug abuse, and diet. Utilizing a SIV-macaque model, we were able to undertake a controlled study of retroviral infection, antiretroviral drug treatment, and their impact on the hepatic immune environment. Based on previous studies and the data presented here, we propose the following model of liver dysfunction during HIV/SIV infection that is initiated by infection of SIV target cells, presumably CD4 T cells, that localize around portal triads. Viral stimulation initiates a CCL2 chemokine gradient resulting in infiltration of inflammatory monocytes/macrophages into the liver as part of the antiviral response. These infiltrating macrophages enhance recruitment of SIV-target cells to the liver through production of CCR5 ligands, such as CCL3, setting up a feedback loop to amplify SIV levels and further increase liver inflammation. The use of cART results in a reduction in this feedback loop where a decrease in SIV burden is associated with lower macrophage levels and a shift in the liver transcriptome that more closely resembles uninfected macaques. However, it is important to note that the inflammatory transcriptional signature was not completely ameliorated during therapy. Overall, these findings provide mechanistic insights into how SIV/HIV infection promotes liver inflammation through macrophage infiltration to result in liver complications that are observed in HIV-infected individuals.

## Materials and methods

### Ethics statement

All animal studies were conducted in accordance with protocols approved by the Center for Infectious Disease Research (protocol DS-05 UW), Washington National Primate Research Center (protocols 4314–01, 4213–02 and 4213–03) (Seattle, WA), and Yerkes National Primate Research Center (protocol YER-2002662) (Atlanta, GA) under Institutional Animal Care and Use Committees (IACUCs). All macaques in this study were managed according to the the laws, regulations, and guidelines set forth by the United States Department of Agriculture, Institute for Laboratory Animal Research, Public Health Service, National Research Council, Centers for Disease Control, the Weatherall Report titled “The use of nonhuman primates in research”, and the Association for Assessment and Accreditation of Laboratory Animal Care (AAALAC) International. The nutritional plan utilized by the WaNPRC and YNPRC consisted of standard monkey chow supplemented with a variety of fruits, vegetables, and other edible objects as part of the environmental enrichment program established by the Behavioral Management Unit. In addition, other means of enrichment were delivered and overseen by veterinary staff with animals having access to more than one category of enrichment. Paired (uninfected) macaques exhibiting incompatible behaviors were managed by the Behavioral Management staff and managed accordingly. SIV-infected macaques were housed in individual, adjoining cages allowing for social interactions. Primate health was monitored daily by trained staff. All efforts were made to minimize suffering through the use of minimally invasive procedures, anesthetics, and analgesics when determined appropriate by veterinary staff. Animals were painlessly euthanized by sedation with ketamine hydrochloride injection followed by intravenous barbiturate overdose in accordance with the recommendations of the panel of euthanasia of the American Veterinary Medical Association.

### Nonhuman primate studies

The liver tissue samples utilized in this study consisted of adult (uninfected N = 4, SIV+ N = 6, SIV+ cART N = 6) and infant (uninfected N = 7, SIV+ N = 9, SIV+ cART N = 4) Indian rhesus macaques (*Macaca mulatta*). All SIV-infected adult macaques were infected intrarectally with SIVMAC239x. Adult macaques receiving cART were started on an ART regimen 130 days post-infection consisting of subcutaneous tenofovir (20 mg/kg body weight) and emtricitabine (30 mg/kg) and oral raltegravir (50 mg twice daily). Samples from uninfected adult macaques were obtained through the tissue donor program from animals undergoing routine necropsy at Washington National Primate Research Center. Infant rhesus macaques (less than 9 months of age) were challenged orally with low doses of SIVmac251. Infants that become infected with SIV were placed in the SIV-infected group while infants remaining uninfected post-challenge were used as uninfected age-matched controls. SIV-infected infant macaques treated with cART were also infected with SIVmac251 and were placed on an ART regimen 35 days post-infection consisting of tenofovir, emtricitabine and dolutegravir administered as a triple formulation in a single daily injection. Liver samples and plasma were obtained from each animal at necropsy. To conduct the various experiments presented throughout this paper, liver tissues were either flash-frozen or formalin-fixed. A summary of SIV clinical parameters for the infected animals utilized in this study is presented below (Table 5).

**Table 5:** Characteristics of SIV-infected macaques.

	Animal	Weeks Post Infection	Weeks on cART	CD4 T cells (cells/uL)	Plasma Viral Load (copies/mL)
**SIV+ Infant Macaques**	A14203	15	-----	1568	22,009,619
	A14206	20	-----	632	300,799
	A14112	19	-----	877	369,654
	A14113	19	-----	1534	61,655,620
	A14114	19	-----	3186	215,235,030
	A14115	17	-----	632	9,179,343
	A15067	16	-----	2862	39,141,398
	A15068	17	-----	1937	26,529
	A15187	27	-----	1520	1,036,566
**SIV+ cART Infant Macaques**	RHi16	42	37	950	< 60
	RSi16	31	26	1176	< 60
	RPi16	36	31	1064	< 60
	RUh16	38	33	979	< 60
**SIV+ Adult Macaques**	A14049	14	-----	543	133,157,690
	A14050	17	-----	388	165,603,120
	Z09064	18	-----	522	18,587,124
	Z09068	18	-----	261	659,381
	Z09086	20	-----	276	655,101
	Z09096	20	-----	392	681,150
**SIV+ cART Adult Macaques**	A12015	54	35	1030	55
	A13273	54	35	237	46
	A13274	54	36	369	< 30
	A13275	54	36	607	192
	A13276	55	36	351	125
	A13277	55	36	510	< 30

### Liver histopathology

Formalin-fixed, paraffin-embedded 5-um liver sections were stained with hematoxylin and eosin (H&E). A board-certified veterinary pathologist with expertise in nonhuman primate pathology reviewed the H&E stained liver sections in a blinded fashion. Findings from the liver pathology report were considered within normal background of macaques, and include vacuolar hepatopathy and lipid accumulation in stellate cells. No significant differences were observed between the treatment groups.

### Immunofluorescence microscopy

Liver tissues obtained at necropsy were fixed in 10% formalin and then paraffin embedded. Tissue sections (5-um) were mounted on glass slides and used for immunofluorescence microscopy. Slides were dewaxed in xylene and rehydrated through graded ethanols into distilled water. Antigen retrieval was performed using Antigen Unmasking Solution (Vector Laboratories, H-3300) in a decloaking chamber (Biocare Medical, Concord, CA) at 90°C for 30 minutes and then cooled for 10 minutes before removing. After washing slides twice for 5 minutes in 0.025% TritonX-100 in 1X TBS, tissue sections were blocked for two hours in 0.1% BSA + 1% goat serum in 0.025% TritonX-100 in TBS. Excess liquid was removed from the tissue section by aspiration and then each section was outlined with a hydrophobic barrier. Sections were incubated overnight at 4°C with specific antibodies for CD68 (clone KP1, Santa Cruz, 1:250) to identify macrophages, CD3 (clone SP7, ThermoFisher Scientific, 1:150) to identify T cells, and CD4 (clone BC/1F6, Abcam, 1:250) to identify CD4 T cells. The following day, slides were washed three times in 0.025% TritonX-100 in TBS for ten minutes each followed by incubation with fluorescent secondary antibodies for one hour at room temperature protected from light. CD68 and CD4 staining were detected using AlexaFluor 594 goat anti-mouse (Life Technologies, 1:500) while CD3 was detected using AlexaFluor 488 goat anti-rabbit (Life Technologies, 1:500). After the one hour incubation, slides were washed three times for five minutes each in 0.025% TritonX-100 in TBS. Slides were then mounted using Vectorshield Hard Set Mounting Medium with Dapi (Vector Laboratories, H1500), cured overnight at 4C, and then imaged the following day on a fluorescent microscope. Each liver section was imaged under 100X magnification in eight random fields of view. Macrophages were quantified using the particle analysis feature in ImageJ software while T cells were enumerated with the cell counting platform in ImageJ. Animals A12015 and A13273 were omitted from the microscopy analysis due to lack of fixed liver specimen.

### Immunohistochemical microscopy

Tissue sections (5-um) were dewaxed in xylene and rehydrated through graded ethanols into distilled water. Antigen retrieval was performed using Antigen Unmasking Solution (Vector Laboratories, H-3300) in a decloaking chamber (Biocare Medical, Concord, CA) at 95°C for 20 minutes. Slides cooled for 20 minutes before removing, and were then placed in water for five minutes followed by wash buffer (0.05% Tween 20 in TBS) for five minutes. Immunohistochemistry was conducted using reagents from the EnVision G2 Doublestain System (Dako, K5361). Slides were incubated in dual endogenous enzyme block for five minutes at room temperatures followed by two washes in wash buffer for five minutes each. Tissues were blocked with 0.25% casein in PBS for 30 minutes at room temperature. Primary antibody, either MAC387 (Abcam ab80084, 1:200) or CCL2 (Invitrogen MA5-17040, 1:500), was added to each slide and incubated for one hour at room temperature. Following two washes for five minutes each in wash buffer, polymer HRP was added for 30 minutes at room temperature, and then two additional wash steps. DAB chromogen (prepared per the manufacturer’s instructions) was added to each slide and the development of color was monitored under the microscope. All slides were stopped at the same time by placing the slides in di-water. Slides were washed one time in wash buffer and then counterstained in CAT Hemotoxylin for 15 seconds. Counterstained sections were rinsed in water until clear and then blued in Scott’s water for 30 seconds. Tissue sections were air-dried overnight and then mounted with Richard Allen Mounting Medium the following day. Images were acquired by Brightfield microscopy.

### Flow cytometry analysis of PBMC and liver cells

PBMC were obtained by ficoll-paque density centrifugation and cryopreserved in 90% FBS-10% DMSO at 10 million cells per mL. Liver cell suspensions were acquired from liver tissue at necropsy by flushing tissue with cold RPMI to remove blood, mincing the tissue into small pieces, and then mashing through a 70 μm cell strainer. Liver cells were pelleted, counted, and then cryopreserved in 90% FBS-10% DMSO at 10 million cells per tube. For flow cytometry analysis, cryopreserved cells were thawed, rested for 1 hour in RPMI at 37°C, 5% CO_2_, and then counted. Two million total cells were used for each stain. CCR2-BV421 clone 48607 (BD Biosciences) was added to each cell suspension and incubated at 37°C for 15 minutes. Next, antibodies for extracellular staining and live/dead staining (Live/Dead Aqua Fixable Dead Stain, Life Technologies) were added and incubated with cells at room temperature for 20 minutes. Extracellular antibodies consisted of CD16-BV605 clone 3G8 (BD Biosciences), CD14-BV785 clone M5E2 (Biolegend), CD11b-PE clone ICRF44 (BD Biosciences), CD45-APC clone MB4-6D6 (Miltenyi), and CD3-APC-H7 clone SP34-2 (BD Biosciences). Cells were washed once in PBS + 2% FBS, pelleted, and then permeabilized in FACS Juice for 10 minutes on ice. Following two washes in PBS + 2% FBS, cells were stained with intracellular antibodies for 20 minutes at room temperature. Intracellular antibodies consisted of CD68-PECy7 clone Y1/82A (BD Biosciences) and S100A9-FITC clone Mac387 (Thermo Fisher). After a final wash in PBS + 2% FBS, cells were resuspended in 1% PFA and acquired on a BD LSRII flow cytometer. Data were analyzed using FlowJo software (version 1.1.0-SNAPSHOT). Gates for cell populations were determined using fluorescence minus one (FMO) stained controls.

### Phagocytosis of E. coli

Liver cells were freshly isolated from liver tissue as described above. Two million liver cells were pelleted by centrifugation and resuspended in 100 uL of pHrodo E. coli bioparticles (Life Technologies, P35361) prepared at 1 mg/mL in PBS + 2% FBS. Cells were incubated with E. coli bioparticles for 2 hours at 37°C, 5% CO_2_. A negative control was conducted using 10 uM cytochalasin D. Cells were washed once with PBS + 2% FBS and then stained with CD3-Pacific Blue clone SP34-2 (BD Biosciences), CD4-BV650 clone OKT4 (Biolegend), CD8-APC-H7 clone SK1 (BD Biosciences), CD45-APC clone MB4-6D6 (Miltenyi), CD14-BV785 clone M5E2 (Biolegend), CD68-FITC clone Y1/B2A (eBioscience), and Live/Dead Aqua Fixable Dead Stain (Life Technologies) for 20 minutes at room temperature. After a wash in PBS + 2% FBS, cells were resuspended in PBS + 2% FBS and acquired on a BD LSRII flow cytometer with phagocytosed E. coli bioparticles detected on the PE filter. Data were analyzed using FlowJo software (version 1.1.0-SNAPSHOT).

### Liver pulverization and nucleic acid isolation

Flash-frozen liver tissues were pulverized using a Retsch Planetary Ball Mill PM100 under cryogenic conditions using liquid nitrogen. To disrupt the tissue and obtain a fine powder homogenate, each tissue was placed in a 50 mL grinding jar with 20 mm stainless steel balls and subjected to 3 cycles of grinding at 300 rpm for 2 minutes each. The tissue powder was then collected into a sterile tube and stored at -80°C. For nucleic acid isolation, ~10 mg of liver powder was used to obtain RNA using the NucleoSpin RNA isolation kit (Macherey-Nagel) or DNA using the NucleoSpin Tissue Genomic DNA Isolation Kit (Macherey-Naglel). Nucleic acid concentrations for RNA and DNA were obtained using a NanoDrop 2000 Spectrophotometer (Thermo Scientific). Isolated nucleic acids were stored at -80°C until use.

### Quantitative RT-PCR (qRT-PCR)

cDNA was prepared from isolated liver RNA (1 ug) using a High-Capacity RNA-to-cDNA kit (Life Technologies) following the manufacturer’s instructions. cDNA was diluted (1:10) in nuclease-free water and used to quantify liver transcript levels of chemokines and cytokines, including TNFα, CCL3 (MIP-1α), TGFβ, IL-10, and CCL2 (MCP-1), and the chemokine receptor, CCR2, using rhesus macaque-specific TaqMan Gene Expression Assays (ThermoFisher Scientific). Glyceraldehyde-3-phosphate dehydrogenase (GAPDH) was used as the housekeeping gene for data normalization. Each 20 uL reaction contained 4 uL of diluted cDNA, 1 uL of TaqMan Gene Expression Assay Mix, 10 uL of Taqman Gene Expression Master Mix, and 5 uL water. PCR reactions were performed on an ABI 7500 detection system (Applied Biosystems) with one cycle at 95°C for 10 minutes followed by 47 cycles of 95°C for 15 seconds and 55°C for one minute. Any sample displaying high standard deviation between duplicates was omitted from downstream analysis. Relative expression of each transcript of interest was determined using the comparative cycle threshold (Ct) method. Genes of interest were normalized to the endogenous control GAPDH mRNA. Relative mRNA expression levels were then determined using the formula 2^-ΔΔCt^ with an uninfected, age-matched control for determination of relative fold change. Data were then Log2 transformed for statistical and correlation analyses.

### Liver oligonucleotide microarray processing

RNA was extracted as described above. RNA samples were then verified for purity, and the quality of the intact RNA was assessed using an Agilent 2100 Bioanalyzer. cRNA probes were made from each sample by Agilent one-color Quick-Amp labeling kit. Each cRNA sample was then hybridized to Agilent Rhesus whole-genome oligonucleotide microarrays (4x44k) based on the manufacturer’s instructions. Slides were scanned with an Agilent DNA microarray scanner, and the output images were then analyzed using Agilent Feature Extractor software. For each microarray, raw intensities, probe mappings, and quality-control (QC) metrics were uploaded into a custom laboratory information management system (LabKey Software).

### Liver microarray data analysis

Raw Agilent Microarray files were extracted using Agilent feature extractor version (version 10.7.3.1). Raw Microarray files were downloaded, background corrected using the “norm-exp” method with an offset of 1 and quantile normalized using the limma bioconductor package in the R statistical software environment (version 3.1.3). Replicate probes were mean summarized, and low expressed probes were removed. Exploratory analysis was performed in R. Statistical analysis was performed through the limma package and differentially expressed gene sets were uploaded into Ingenuity Pathway Analysis for Functional Analysis (IPA). Raw and Normalized expression data was submitted to the GEO (accession GSE97676). Gene expression profiles in SIV-infected and SIV-infected cART macaques were compared to age-matched, uninfected macaque expression data using limma. Differentially expressed genes were defined as having greater than 1.5-fold change over uninfected macaques with a Benjamini-Hochberg corrected (adjusted p value) less than 0.05. Co-expression analysis was performed using packages WGCNA and heapmap.2 in R/Bioconductor.

### Luminex analysis

Plasma was obtained by centrifugation (1,000 x g, 10 minutes) from blood collected at necropsy and stored at -80°C until use. Levels of circulating immune-associated factors were determined using a custom 20plex Nonhuman Primate ProcartaPlex Multiplex Immunoassay (eBioscience). Assayed analytes include: IFNα, MIP-1α, IFNγ, IL-1β, TNFα, IL-1RA, IL-10, IL-12p70, IL-17A, IL-18, IL-23, IL-4, IL-6, IL-7, IL-8, IP-10, MCP-1 (CCL2), MIG, MIP-1β, sCD40L. Plasma from cART-treated infants was unavailable for analysis. Each plasma sample was assayed in duplicate according to the manufacturer’s instructions on a Bio-Plex 200 (BioRad, Hercules, California). Plasma concentration for each analyte was calculated from a standard curve using a five-parameter logistic regression after background subtraction. Analytes below the detection of limit were recorded as zero for correlation analyses.

### Plasma viral loads

Plasma viral loads were determined for all SIV-infected macaques at the necropsy time-point by real-time reverse-transcription PCR (RT-PCR) based on methods originally described by Suryanarayana et al. [105]. Briefly, plasma purified viral RNA was amplified using oligonucleotides for SIVgag in conjunction with a TaqMan-based probe. Viral load (copies/mL) was determined from a standard curve.

### Quantification of bacterial 16S DNA in the liver

Levels of bacterial 16S DNA in the liver was assessed by qPCR using a Femto Bacterial DNA Quantification Kit (Zymo Research, E2006) per the manufacturer’s instructions. Briefly, total DNA was extracted as described above and diluted in nuclease-free water to a final concentration of 100 ng/uL. Each reaction contained 1 uL of total liver DNA (100 ng) and 18 uL of Femto Bacterial qPCR Premix. A ‘No Template’ Negative Control was also conducted to test for any possible contamination of qPCR reagents. PCR reactions were performed on an ABI 7500 detection system (Applied Biosystems) with one cycle at 95°C for 10 minutes followed by 40 cycles of 95°C for 30 seconds, 55°C for 30 seconds, and 72°C for one minute. One final extension step was performed at 72°C for seven minutes. A standard curve was generated with bacterial DNA standards (supplied with the kit) ranging from 0.00002 to 20 ng of bacterial DNA (R^2^ = 0.9576). The concentration of bacterial DNA in each liver sample was determined from the standard curve using a nonlinear regression four-parameter variable slope analysis. Duplicates were averaged for each animal and plotted as ng of 16S bacterial DNA per 100 ng of total input DNA.

### Quantification of LBP in plasma

LPS-binding protein (LBP) was quantified in the plasma of each macaque using an ELISA kit from Biometic (ABIN370809) per the manufacturer’s instructions. Absorbance was read at 450 nm using a Molecular Devices SpectraMax M2 plate reader. Any sample displaying absorbance below background was excluded from downstream analysis. A standard curve was generated (R^2^ = 0.999) and used to determine the concentration of LBP in each plasma sample assayed in duplicate.

### Quantification of SIV DNA in the liver

Assessment of SIV DNA in the liver was measured by quantitative hybrid real-time/digital RT-PCR [52, 53]. For each liver sample, ten replicate reactions were run. Animal A13277 (SIV-infected cART adult) was omitted from this analysis due to lack of starting material for DNA isolation. Quantitation of samples showing positive amplification in all ten replicates was determined directly from a standard curve. For samples that did not have positive amplification in all ten replicates, SIV DNA quantification was determined from the frequency of positive amplifications, corresponding to the presence of at least one target copy in a reaction, according to a Poisson distribution of a given median copy number per reaction. Cell number was determined by qPCR for a single copy sequence from the rhesus macaque CCR5 gene and then used to normalize SIV DNA copies per 10^6^ diploid genome equivalents. For three samples (1.2–2.7 X 10^5^ diploid genome equivalents analyzed), SIV DNA was not detected, corresponding to a nominal assay threshold of less than 4–9 copies/10^6^ cell equivalents.

### RNAscope analysis

In situ hybridization analysis was conducted using RNAscope technology (Advanced Cell Diagnostics) to identify SIV viral RNA-positive cells in the liver. Target probes, complementary to the SIV plus-RNA strand were designed to hybridize to SIV viral RNA in gag, pol, tat, env, vpx, vpr, nef, and rev genes. Upon binding, a pair of probes forms a double Z configuration and allows for signal amplification, followed by the chromogenic detection of SIV-RNA+ cells using a horseradish peroxidase enzymatic reaction. Liver sections (5-um) on glass slides were baked for one hour at 60°C and then placed in xylene (2 x 5 minutes) followed by 100% ethanol (2 x 3 minutes). Endogenous peroxidases were quenched using RNAscope hydrogen peroxide reagent (catalog no. 322335) for 10 minutes at room temperature followed by one wash in di-water. Antigen retrieval was performed in boiling 1x antigen retrieval buffer (catalog no. 322001) diluted in di-water for 30 minutes, followed by three quick washes in di-water and 1 minute in 100% ethanol. Sections were then air dried at room temperature (~ five minutes) and then outlined with a hydrophobic barrier. Protease III reagent (catalog no. 322331) was utilized at a 1:3 dilution in sterile PBS and incubated on the tissue sections at 40°C in the HybEZ hybridization oven (Advanced Cell Diagnostics) for 20 minutes followed by two rinses in di-water. SIVmac239 target probes (catalog no. 416141) were incubated on the tissue for two hours at 40°C in the HybEZ hybridization oven, followed by two washes in the RNAscope wash buffer (catalog no. 322000) for two minutes each. Signal amplification was conducted at 40°C in the HybEZ oven at the following conditions: Amp1 (catalog no. 322501) for 30 minutes, Amp2 (catalog no. 322502) for 15 minutes, Amp3 (catalog no. 322503) for 30 minutes, and Amp4 (catalog no. 322504) for 15 minutes. After each amplification step, slides were washed two times in RNAscope wash buffer for two minutes before proceeding to the next amplification step. The remaining amplification steps, Amp5 (30 minutes, catalog no. 322509) and Amp6 (15 minutes, catalog no. 322510) were conducted at room temperature with washing using RNAscope Wash Buffer conducted between the steps. Following amplification, signal was developed using DAB-A and DAB-B (mixed in a 1:1 ratio) on the liver sections for two minutes at room temperature followed by two washes in RNAscope Wash Buffer for two minutes each. Slides were then counterstained in hematoxylin for 30 seconds and rinsed with tap water until clear. Tissues were cleared in Scott’s Water for 30 seconds, then dehydrated through graded ethanols and xylene, and mounted using Permount mounting media. Whole liver sections were imaged by a Nanozoomer Scanner (Hamamatsu Photonics) and then analyzed by NDP Viewer software (Hamamatsu Photonics) under 5x and 20x magnification.

For phenotyping SIV RNA-positive cells, TSA Cy3.5 (Perkin Elmer, NEL76300), prepared 1:500 in Amplification Diluent, was substituted for the DAB-A and DAB-B reagent following the amplification steps. After seven minutes of incubation, TSA Cy3.5 treated slides were rinsed in di-water for 10 minutes, then blocked in 4% normal goat serum + 0.25% casein in 1X TBS with 0.05% Tween 20 (TBS-Tween) for 15 minutes. Mouse anti-CD68 clone KP1 (1:200, Santa Cruz) and rabbit anti-CD3 clone SP7 (1:100, ThermoFisher Scientific) were added to TSA Cy3.5 developed slides overnight at 4°C. The following day, slides were rinsed in TBS-Tween wash buffer, and then incubated with goat anti-mouse Alexa647 and goat anti-rabbit Alexa488 (both at 1:200, ThermoFisher Scientific) for one hour at room temperature. Following one wash in TBS-Tween, slides were incubated in Sudan Black for 15 minutes to reduce tissue auto-fluorescence. Slides were then washed one more time in TBS-Tween buffer, counterstained in DAPI (1 ug/mL) for 5 minutes and then mounted using Prolong Gold Mounting Medium (ThermoFisher Scientific). Images were acquired on a Nikon fluorescent microscope under 600x magnification.

### Isolation, generation, and in vitro stimulation of human monocyte-derived macrophages

Human PBMC were isolated by ficoll-paque density centrifugation from blood obtained from a healthy donor. Monocytes were isolated by negative selection using a Pan Monocyte Isolation Kit (Miltenyi). Isolated monocytes were counted and resuspended at 2 million cells/mL in RPMI containing 10% FBS, Penstrep, and 50ng/mL recombinant human GM-CSF (Peptrotech, 300–03). Mononcytes were plated in 24-well plates at 1 million cells/well (500 uL/well), and differentiated into macrophages over the course of seven days in the presence of GM-CSF (50 ng/mL). On day 3 and 5, the culture media was removed and replenished with fresh media containing GM-CSF. Any unattached cells were removed during feeding. On day 7, the culture media containing GM-CSF was removed and replaced with RMPI + 10% FBS and Penstrep (complete RPMI). Macrophages were rested for 24 hours, and then stimulated with either Poly I:C HMW (Invivogen, tlrl-pic) or ssRNA40 (Invivogen, tlrl-lrna40) at 0.2 and 2 ug/mL in duplicate for 12 hours. Unstimulated control macrophages received only complete RPMI media. After 12 hours, the supernatant was removed and macrophages were rinsed once with warm PBS + 2% FBS followed by lysis in 350uL of RA1 buffer containing 1% beta-mercaptoethanol. Cell lysates were stored at -80°C until RNA extraction. RNA was prepared using the NucleoSpin RNA isolation kit (Macherey-Nagel) and quantified using a NanoDrop 2000 Spectrophotometer (Thermo Scientific). One microgram of RNA was used to prepare cDNA using a High-Capacity RNA-to-cDNA kit (Life Technologies) following the manufacturer’s instructions. cDNA was diluted (1:10) in nuclease-free water and used to quantify transcript levels of CCL2, CCL3, TNFα, TGFβ and GAPDH using human-specific TaqMan Gene Expression Assays (ThermoFisher Scientific). Each 20 uL reaction contained 4 uL of diluted cDNA, 1 uL of TaqMan Gene Expression Assay Mix, 10 uL of Taqman Gene Expression Master Mix, and 5 uL water. PCR reactions were performed on an ABI 7500 detection system (Applied Biosystems) with one cycle at 95°C for 10 minutes followed by 47 cycles of 95°C for 15 seconds and 55°C for one minute. Relative expression of each transcript of interest was determined using the comparative cycle threshold (Ct) method. Genes of interest were normalized to the endogenous control GAPDH mRNA. Relative mRNA expression levels were then determined using the formula 2^-ΔΔCt^ with unstimulated macrophages used for determination of relative fold change. Statistical significance was determined using Mann Whitney T tests comparing technical replicates of stimulated macrophages to unstimulated macrophages.

### Statistics

Statistical analyses were performed using Prism version 5.0f software (GraphPad Software, Inc., San Diego, CA). A Mann-Whitney nonparametric U test was used to compare all groups. For correlation analyses, data were first assessed for normality using D’Agostino & Pearson omnibus normality test. Unless normality was violated, Pearson Correlation analysis was conducted. Alternatively, Spearman Correlation was conducted for data not displaying normality.

## Supporting information

S1 Fig **A)** Immunofluorescence microscopy for CD3 T cells (green) and CD68 macrophages (red) around the portal triad of a SIV-infected macaque (scale bar = 100 um). **B)** Immunofluorescence microscopy to determine the phenotype of T cells around the portal triad by double staining for CD3 (green) and CD4 (red) (scale bar = 35 um).
(TIFF)

S2 Fig Age-related differences in IL-10. cDNA was prepared from RNA isolated from the liver and used to evaluate liver IL-10 gene expression while serum IL-10 levels were determined by Luminex. **A)** Differences in IL-10 expression in the liver between treatment groups with adult macaques in open symbols and infant macaques in closed symbols. **B)** Differences in liver IL-10 expression between infants and adults were determined by normalizing SIV+ and SIV+ ART expression levels to the average expression in age-matched uninfected macaques. **C)** Circulating levels of IL-10 in the blood as measured by Luminex determined higher IL-10 levels in infants when compared to adults.
(TIFF)

S3 Fig Antiviral signature in the liver of SIV-infected infant macaques. Ingenuity Pathway Analysis for Functional Analysis (IPA) found gene signatures in the liver of SIV-infected macaques compared to uninfected macaques known to be involved in antiviral defense. **A)** Evaluation of the canonical interferon signaling pathway indicates that several genes (shaded in red) are significantly (p < 0.05) upregulated at least 1.5-fold. Many of these genes are involved in signal transduction (e.g. STATs) or are downstream antiviral effector interferon-stimulated genes (ISGs) (e.g. OAS1, IFIT, IRFs). Genes that show activity, but do not meet the p value or fold change criteria are outlined in gray. **B)** Antiviral network analysis showing the drivers (depicted in red) of the liver antiviral response in SIV-infected macaques.
(TIFF)

S4 Fig Antiviral signature in the liver of SIV-infected adult macaques. Ingenuity Pathway Analysis for Functional Analysis (IPA) found gene signatures in the liver of SIV-infected macaques compared to uninfected macaques known to be involved in antiviral defense. **A)** Evaluation of the canonical interferon signaling pathway indicates that some genes (shaded in red) are significantly (p < 0.05) upregulated at least 1.5-fold. Many of these genes are downstream antiviral effector interferon-stimulated genes (ISGs) (e.g. OAS1, Mx1, IRFs). Genes that show activity, but do not meet the p value or fold change criteria are outlined in gray. **B)** Inflammatory network analysis showing the drivers (depicted in red) of the liver antiviral response in SIV-infected macaques.
(TIFF)
