# Supplementary figures and images for: Liver macrophage-associated inflammation correlates with SIV burden and is substantially reduced following cART
Source: PLoS Pathog. 2018 Feb 21;14(2):e1006871. doi: 10.1371/journal.ppat.1006871 (PMC5837102; doi:10.1371/journal.ppat.1006871)

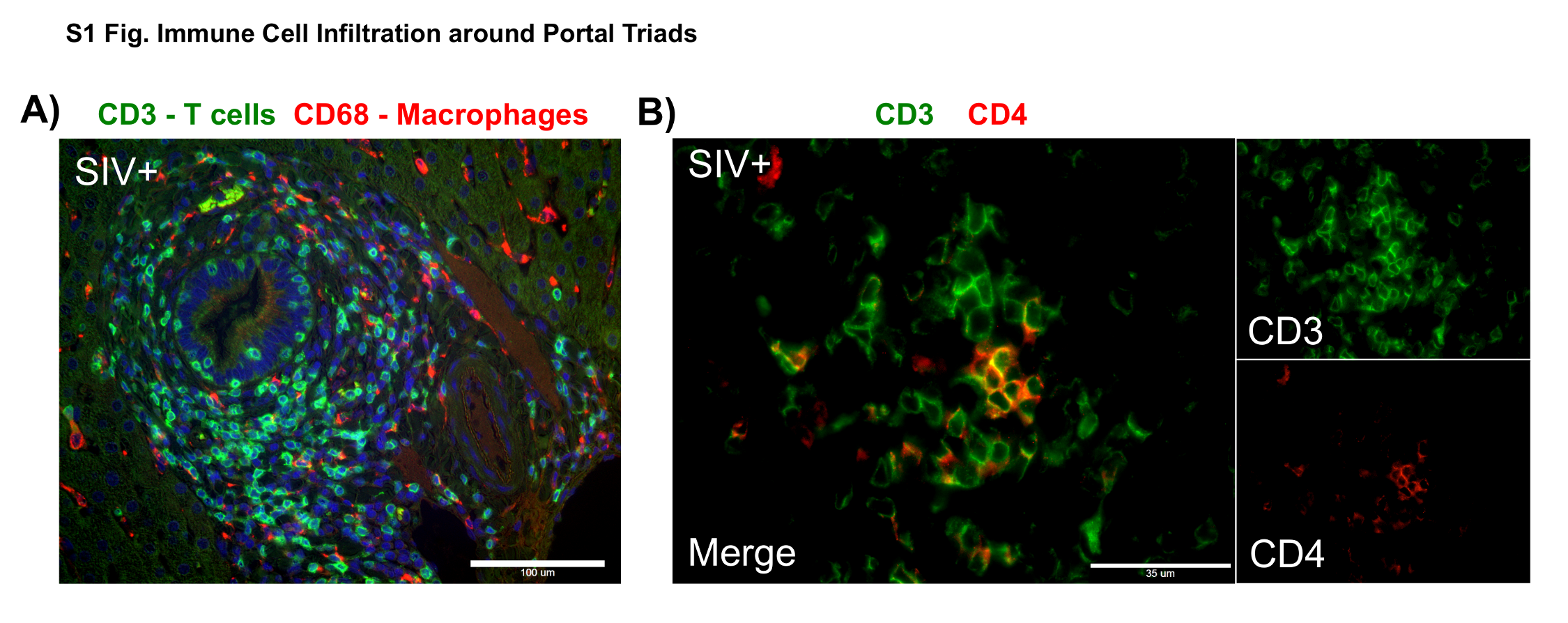

Supplement: S1 Fig — A) Immunofluorescence microscopy for CD3 T cells (green) and CD68 macrophages (red) around the portal triad of a SIV-infected macaque (scale bar = 100 um). B) Immunofluorescence microscopy to determine the phenotype of T cells around the portal triad by double staining for CD3 (green) and CD4 (red) (scale bar = 35 um). (TIFF) [file ppat.1006871.s001.tiff]

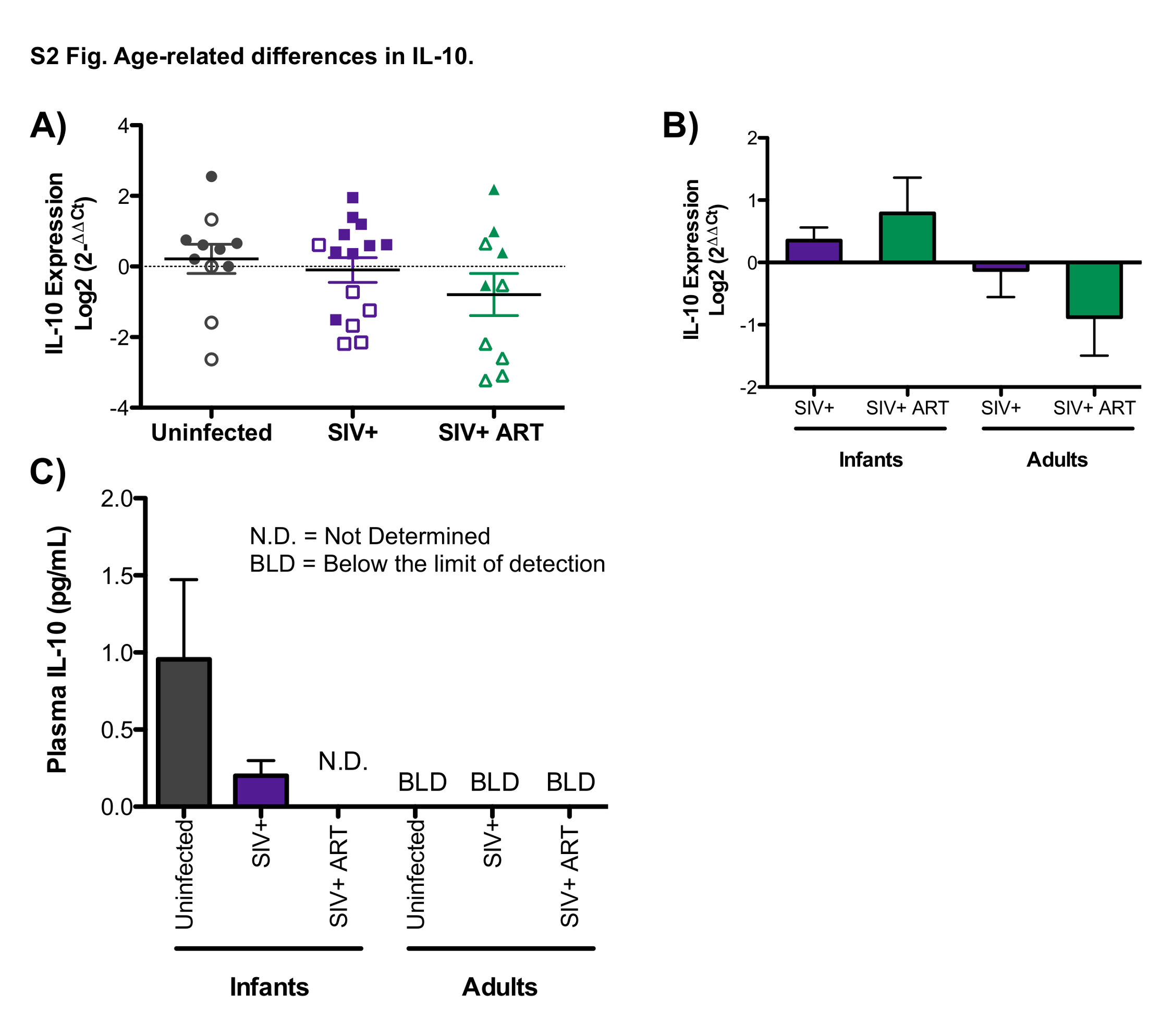

Supplement: S2 Fig — cDNA was prepared from RNA isolated from the liver and used to evaluate liver IL-10 gene expression while serum IL-10 levels were determined by Luminex. A) Differences in IL-10 expression in the liver between treatment groups with adult macaques in open symbols and infant macaques in closed symbols. B) Differences in liver IL-10 expression between infants and adults were determined by normalizing SIV+ and SIV+ ART expression levels to the average expression in age-matched uninfected macaques. C) Circulating levels of IL-10 in the blood as measured by Luminex determined higher IL-10 levels in infants when compared to adults. (TIFF) [file ppat.1006871.s002.tiff]

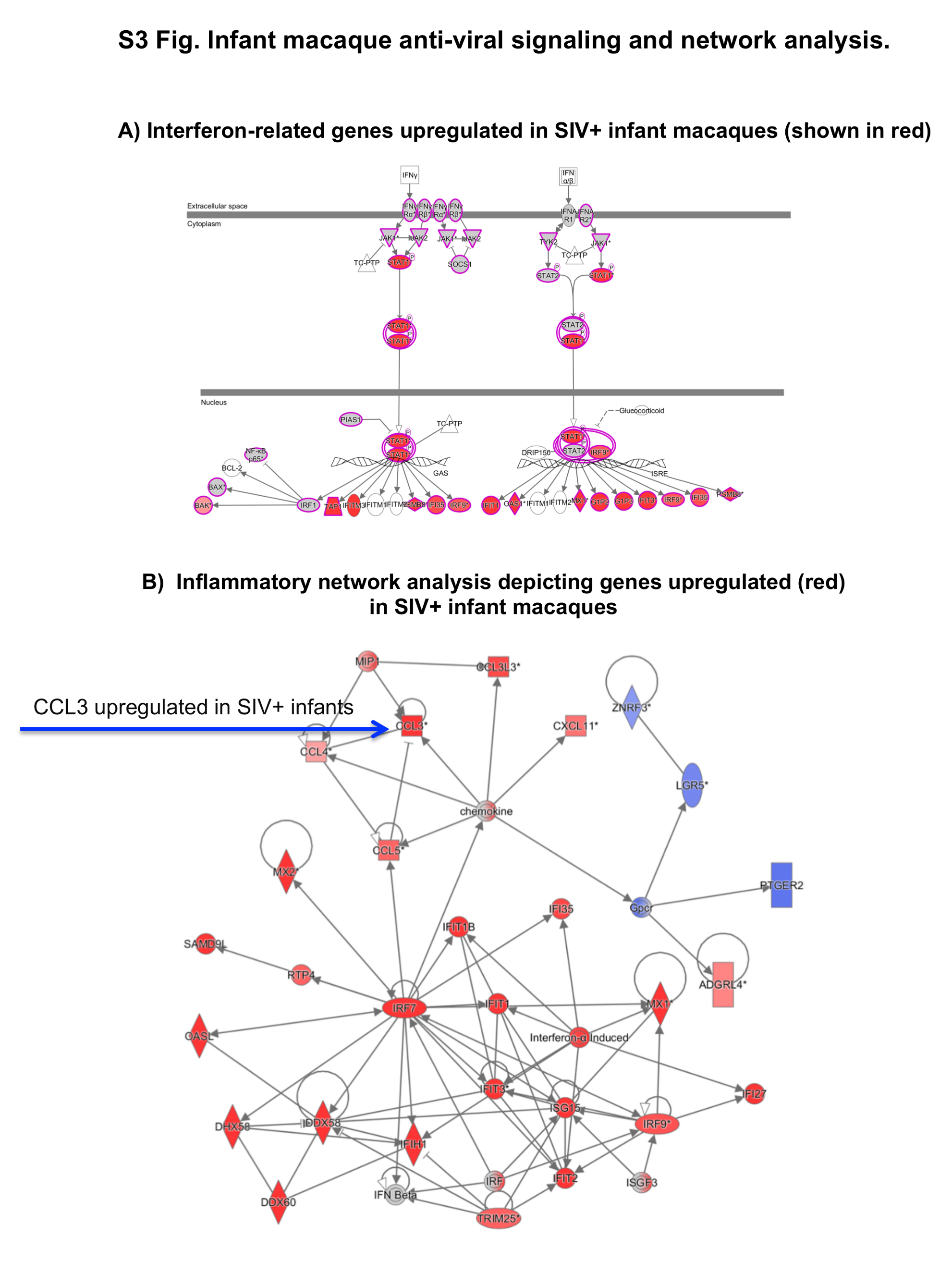

Supplement: S3 Fig — Ingenuity Pathway Analysis for Functional Analysis (IPA) found gene signatures in the liver of SIV-infected macaques compared to uninfected macaques known to be involved in antiviral defense. A) Evaluation of the canonical interferon signaling pathway indicates that several genes (shaded in red) are significantly (p < 0.05) upregulated at least 1.5-fold. Many of these genes are involved in signal transduction (e.g. STATs) or are downstream antiviral effector interferon-stimulated genes (ISGs) (e.g. OAS1, IFIT, IRFs). Genes that show activity, but do not meet the p value or fold change criteria are outlined in gray. B) Antiviral network analysis showing the drivers (depicted in red) of the liver antiviral response in SIV-infected macaques. (TIFF) [file ppat.1006871.s003.tiff]

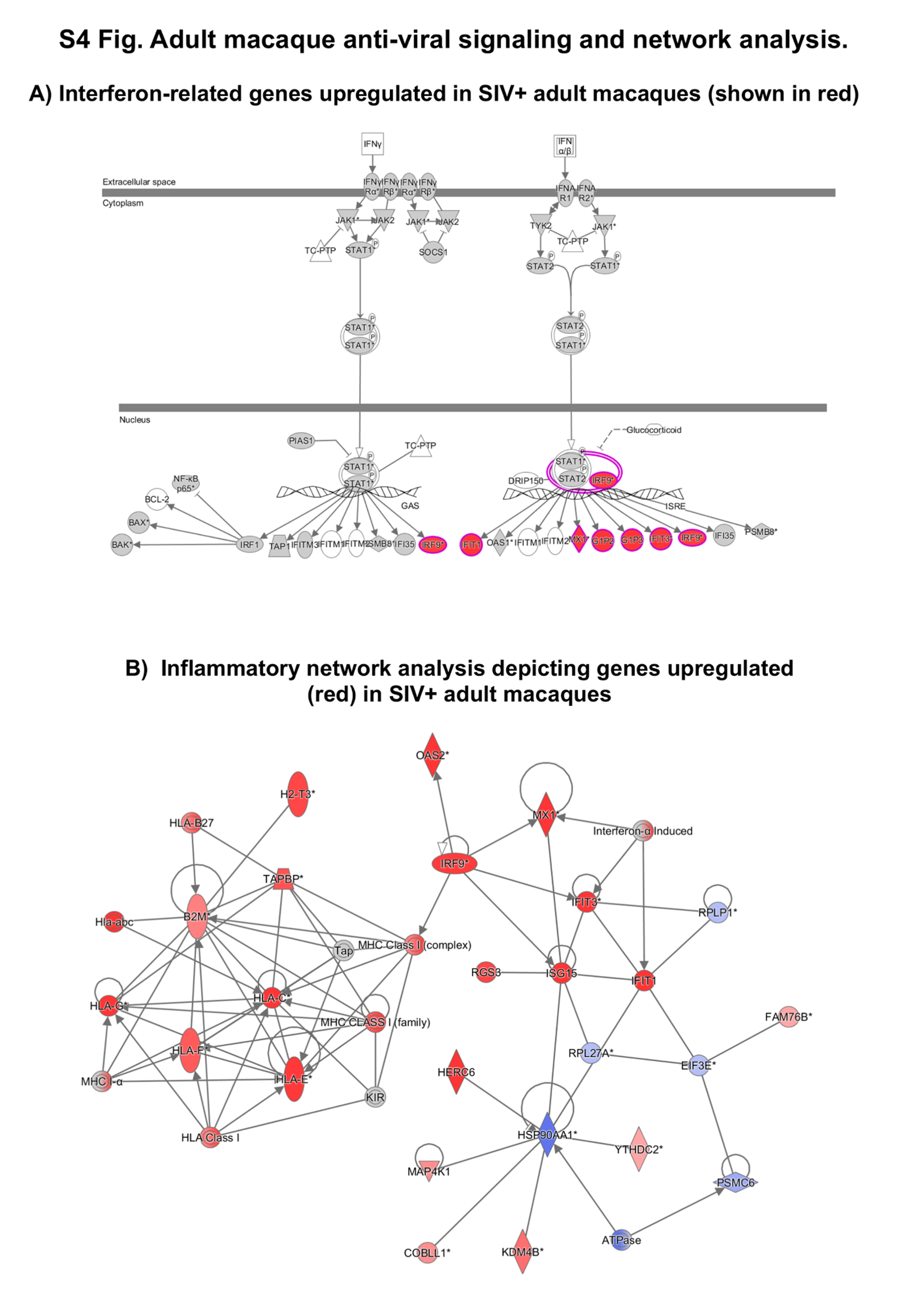

Supplement: S4 Fig — Ingenuity Pathway Analysis for Functional Analysis (IPA) found gene signatures in the liver of SIV-infected macaques compared to uninfected macaques known to be involved in antiviral defense. A) Evaluation of the canonical interferon signaling pathway indicates that some genes (shaded in red) are significantly (p < 0.05) upregulated at least 1.5-fold. Many of these genes are downstream antiviral effector interferon-stimulated genes (ISGs) (e.g. OAS1, Mx1, IRFs). Genes that show activity, but do not meet the p value or fold change criteria are outlined in gray. B) Inflammatory network analysis showing the drivers (depicted in red) of the liver antiviral response in SIV-infected macaques. (TIFF) [file ppat.1006871.s004.tiff]
